# The global prevalence of complete hearing loss in 204 countries and territories from 1992 to 2021: a systematic analysis for the global burden of disease study 2021

**DOI:** 10.3389/fpubh.2025.1526719

**Published:** 2025-04-09

**Authors:** Guan-Jiang Huang, Zhi-Jun Fan, Biao-Qing Lu

**Affiliations:** Department of Otorhinolaryngology Head and Neck Surgery, Zhongshan Hospital of Traditional Chinese Medicine, Affiliated to Guangzhou University of Chinese Medicine, Zhongshan, Guangdong, China

**Keywords:** hearing loss, aging, global burden of disease, trend, prediction

## Abstract

**Background:**

Complete hearing loss, especially the age-related type, poses a significant public health challenge globally. This study aims to assess the global burden on the prevalence of complete hearing loss from 1992 to 2021 and forecast trends up to 2036.

**Methods:**

Using data from the Global Burden of Disease (GBD) Study 2021, we assessed the global burden of complete hearing loss across 204 countries and territories. We analyzed temporal trends in ASPR using Joinpoint regression, evaluated the contributions of age, period, and cohort effects through Age-Period-Cohort modeling, and performed decomposition analysis to determine the impact of demographic and epidemiological changes on prevalence trends. Predictions of future ASPR trends were made using Bayesian Age-Period-Cohort (BAPC) and Autoregressive Integrated Moving Average (ARIMA) models.

**Results:**

By 2021, the global prevalence of complete hearing loss had reached 9.9 million cases, with the ASPR declining from 134.35 to 117.79 per 100,000. The overall Estimated Annual Percentage Change (EAPC) was−0.45. The most significant reductions were observed in low-SDI regions, particularly Sub-Saharan Africa (EAPC: −0.74). In contrast, high-SDI regions, including North America and Western Europe, showed more modest declines (EAPC: −0.18). Notably, East Asia exhibited a 62.3% increase in prevalence, with high-income Asia Pacific showing the highest relative rise at 83.97%. Age-related hearing loss remained the dominant cause, especially among individuals aged 60 and above. Males were more affected than females. Population aging and growth were the major drivers of the increased prevalence in high-SDI regions, while population growth was the primary factor in low-SDI areas.

**Conclusion:**

The burden of complete hearing loss remains high in prevalence, particularly in aging populations within high-SDI regions, despite overall reductions in ASPR. Significant regional disparities remain, highlighting the need for targeted interventions to improve access to hearing care and affordable technologies in low-SDI regions.

## Introduction

Complete hearing loss is the most severe hearing impairment, which significantly impacts communication abilities, quality of life, and overall well-being. Hearing loss is a global public health issue that affects millions of people worldwide, with profound implications for individuals and societies (1–3). The etiology of complete hearing loss is multifactorial, involving genetic, environmental, and lifestyle factors (3). Age-related hearing loss, also known as presbycusis, is the most common cause and typically affects individuals over the age of 60 (4). Recent advancements in cochlear implants and hearing aids have provided some relief to those affected. However, challenges remain, particularly in low-and middle-income countries where access to healthcare and assistive technologies is limited (2, 5, 6). Moreover, the stigma associated with hearing loss often leads to social isolation and mental health issues, exacerbating the burden on individuals and healthcare systems (7, 8).

Globally, the distribution of hearing loss is uneven, with significant disparities between regions and socio-demographic groups. High-income countries generally report lower prevalence rates due to better access to healthcare and early intervention programs (9). In contrast, low-and middle-income countries bear a disproportionate burden of hearing loss, driven by factors such as poor healthcare infrastructure, lack of awareness, and higher exposure to risk factors (10). The Global Burden of Disease (GBD) study highlights these disparities, revealing that regions like Sub-Saharan Africa and South Asia have some of the highest rates of hearing loss, while North America and Europe have comparatively lower rates (11). These findings underscore the need for targeted public health interventions to address the growing burden of hearing loss globally.

Using data from the GBD study, this study aims to assess the global burden of complete hearing loss from 1992 to 2021, focusing on age-related causes, and forecast trends up to 2036.

## Materials and methods

### Data source

This study utilized data from the GBD Study 2021^1^, which offers comprehensive epidemiological estimates for a wide array of diseases and injuries across 204 countries and territories. The GBD database, maintained by the Institute for Health Metrics and Evaluation (IHME), provides detailed information on disease prevalence, incidence, and mortality, stratified by age, sex, and location from 1990 to 2021 (11, 12). For this analysis, we focused on the prevalence of complete hearing loss, examining data from 1992 to 2021 to understand temporal trends and geographic variations in disease burden. The study also utilized the Socio-Demographic Index (SDI) to stratify countries and regions, facilitating an analysis of the relationship between socio-economic development and prevalence of complete hearing loss. The SDI data are available for download on IHME Websites^2^.

### Statistical analysis

The prevalence of complete hearing loss was assessed in terms of the age-standardized prevalence rate (ASPR) per 100,000 population. We calculated ASPR for the years 1992 and 2021 to examine changes over this period. The temporal trends in ASPR were analyzed using the Estimated Annual Percentage Change (EAPC). EAPC was calculated for the global population, as well as stratified by gender, SDI quintiles, and geographical regions (13). Joinpoint regression analysis was utilized to identify significant shifts in the trends of ASPR over time. This method pinpointed specific points where the trend notably changed, allowing for the calculation of the annual percentage change (APC) within each identified segment (14). To explore the effects of age, period, and birth cohort on the prevalence of complete hearing loss, we employed an Age-Period-Cohort model. This approach was used to distinguish the independent effects of aging, temporal trends, and generational differences on hearing loss prevalence (15). To predict the future trends in ASPR from 2022 to 2036, we applied the Bayesian Age-Period-Cohort (BAPC) model and the Autoregressive Integrated Moving Average (ARIMA) model (16–18).

To quantify the contributions of population growth, aging, and epidemiological changes to the observed trends on prevalence of complete hearing loss, we conducted a decomposition analysis. This analysis disaggregates the overall change in prevalence into components attributable to demographic and epidemiological factors (19). The decomposition was performed across different SDI quintiles to assess how socio-economic development influenced these factors. Health inequality in prevalence of complete hearing loss was assessed using the slope index of inequality (SII) and concentration curves (3, 20). SII measures the absolute difference in prevalence between the highest and lowest socio-economic groups, while concentration curves depict the cumulative distribution of prevalence relative to SDI rank. These methods were used to evaluate changes in the distribution of hearing loss burden from 1992 to 2021, particularly in relation to socio-economic disparities.

We employed geospatial mapping techniques to visualize the global distribution of ASPR and EAPC, highlighting regions with high and low burdens of hearing loss. In addition, we analyzed the prevalence of hearing loss across different age groups and genders, with a focus on identifying demographic patterns and trends. Bar charts and line graphs were generated to depict these relationships, providing a comprehensive view of the factors influencing hearing loss prevalence.

Joinpoint regression analysis was conducted using Joinpoint software (version 5.2.0)^3^. Except the Joinpoint regression analysis, other statistical analyses were conducted using R software (version 4.4.1)^4^, with specific packages (such as “Joinpoint,” “Epi,” “BAPC,” “INLA,” etc.) utilized for the respective analyses. *p* < 0.05 suggests statistically significant.

## Results

### ASPR in 1992 and 2021 and its temporal trends from 1992 to 2021

By 2021, the global prevalence of complete hearing loss had increased to nearly 9.9 million cases, with an ASPR of 117.79 per 100,000 population, which represents a slight decrease from the 134.35 per 100,000 recorded in 1992. The overall EAPC was −0.45. Notably, the trend differed between males and females, with males experiencing a more pronounced reduction in ASPR (from 131.36 to 114.03 per 100,000) compared to females (from 136.51 to 120.78 per 100,000) (Table 1). The EAPC for males was −0.48, slightly higher than the −0.42 observed for females. When examining SDI levels, significant disparities emerged (Table 1). Low SDI regions showed a marked decline in ASPR, from 203.52 to 167.32 per 100,000, with an EAPC of −0.74. Low-middle SDI regions experienced a more pronounced decline with an EAPC of −0.93. In contrast, high SDI regions saw a more modest decrease in ASPR (from 112.41 to 105.35 per 100,000) with an EAPC of −0.18, indicating that while improvements have been made, they have been less substantial compared to lower SDI regions. Geographically, the burden of complete hearing loss also varied significantly across 21 regions (Table 1). For instance, North Africa and the Middle East witnessed a substantial decrease in ASPR from 277.9 to 192.41 per 100,000, with a striking EAPC of −1.31. Moreover, in high-income areas such as North America and the Asia Pacific, the decrease in ASPR was modest, with EAPCs of −0.33 and −0.33, respectively, (Table 1).

**Table 1:** The cases of prevalence and ASPR of complete hearing loss in 1992 and 2021, and its temporal trends from 1992 to 2021.

	1992		2021		1992–2021
Characteristics	Cases of prevalence (95% UI)	ASPR per 100,000 (95% UI)	Cases of prevalence (95% UI)	ASPR per 100,000 (95% UI)	EAPC (95% CI)
Global	5,919,320 (4,581,762, 7,275,263)	134.35 (106.53, 163.06)	9,868,486 (7,755,679, 12,122,326)	117.79 (92.81, 144.17)	−0.45 (−0.47 – −0.43)
Male	2,733,795 (2,095,867, 3,355,109)	131.36 (104.13, 159.68)	4,503,609 (3,519,720, 5,561,262)	114.03 (90, 139.87)	−0.48 (−0.5 – −0.46)
Female	3,185,526 (2,489,820, 3,894,475)	136.51 (108.03, 165.62)	5,364,876 (4,220,715, 6,583,256)	120.78 (95.21, 147.36)	−0.42 (−0.44 – −0.4)
Low SDI	599,870 (465,888, 730,674)	203.52 (162.92, 245.53)	1,088,284 (838,487, 1,332,132)	167.32 (133.42, 203.71)	−0.74 (−0.8 – −0.69)
Low-middle SDI	1,194,014 (921,970, 1,467,369)	158.29 (126.08, 193.16)	1,865,244 (1,442,619, 2,284,648)	121.34 (95.9, 148.19)	−0.93 (−1.03 – −0.83)
Middle SDI	1,669,394 (1,275,844, 2,056,851)	134.82 (106.46, 163.74)	2,938,124 (2,280,139, 3,667,016)	113.59 (89.12, 139.95)	−0.56 (−0.64 – −0.48)
High-middle SDI	1,210,743 (941,167, 1,486,999)	118.11 (93.11, 143.68)	1,968,336 (1,536,182, 2,462,304)	109.02 (85.53, 134.03)	−0.26 (−0.29 – −0.23)
High SDI	1,240,295 (974,069, 1,527,710)	112.41 (88.79, 137.82)	2,001,417 (1,549,119, 2,493,858)	105.35 (83.2, 129.89)	−0.18 (−0.2 – −0.17)
Andean Latin America	17,313 (13,240, 21,145)	71.73 (56.3, 87.33)	36,011 (27,935, 44,371)	60.16 (46.61, 74.67)	−0.52 (−0.6 – −0.45)
Australasia	28,465 (22,461, 34,886)	118.72 (93.63, 145.3)	54,603 (41,839, 69,217)	107.7 (83.53, 135.13)	−0.36 (−0.51 – −0.21)
Caribbean	18,098 (14,050, 22,099)	63.71 (49.64, 78.57)	29,275 (22,850, 36,575)	55.19 (42.95, 68.72)	−0.51 (−0.59 – −0.43)
Central Asia	57,334 (44,202, 71,078)	112.35 (87.62, 137.53)	81,344 (62,151, 101,041)	101.19 (78.9, 124.41)	−0.41 (−0.48 – −0.35)
Central Europe	150,862 (115,816, 187,999)	106.09 (82.43, 130.42)	184,680 (142,015, 231,324)	92.06 (71.95, 113.68)	−0.52 (−0.59 – −0.45)
Central Latin America	68,923 (52,914, 86,243)	68.98 (53.91, 85.36)	143,323 (110,421, 178,164)	58.11 (44.95, 72)	−0.6 (−0.69 – −0.52)
Central Sub-Saharan Africa	56,694 (43,767, 69,350)	186.14 (148.52, 226.1)	112,632 (85,182, 139,661)	157.85 (123.44, 192.32)	−0.62 (−0.74 – −0.5)
East Asia	1,248,522 (949,690, 1,559,883)	130.4 (102.64, 160.55)	2,390,692 (1,832,496, 3,029,494)	119.17 (93.78, 148.91)	−0.22 (−0.3 – −0.14)
Eastern Europe	287,161 (219,266, 357,581)	108.43 (84.49, 132.52)	309,623 (238,835, 384,488)	96.59 (75.73, 118.29)	−0.44 (−0.52 – −0.37)
Eastern Sub-Saharan Africa	231,826 (181,350, 280,279)	225.98 (179.02, 272.82)	446,951 (344,135, 544,017)	202.14 (160.28, 246.79)	−0.6 (−0.77 – −0.42)
High-income Asia Pacific	196,307 (151,243, 245,614)	96.06 (74.43, 119.31)	382,772 (292,303, 490,688)	86.17 (66.96, 107.32)	−0.33 (−0.48 – −0.18)
High-income North America	601,397 (471,142, 747,940)	171.04 (132.96, 211.27)	919,614 (713,360, 1,159,541)	155 (121.62, 192.31)	−0.33 (−0.37 – −0.3)
North Africa and Middle East	661,219 (514,523, 827,179)	277.9 (218.6, 337.88)	994,749 (762,272, 1,217,123)	192.41 (151.07, 234.14)	−1.31 (−1.34 – −1.28)
Oceania	4,406 (3,328, 5,507)	118.17 (92.48, 144.05)	9,218 (6,973, 11,590)	108.21 (84.89, 132.22)	−0.33 (−0.45 – −0.21)
South Asia	1,021,822 (790,965, 1,253,449)	148.5 (118.05, 180.44)	1,698,859 (1,314,688, 2,097,356)	111.35 (87.92, 136.28)	−0.98 (−1.09 – −0.87)
Southeast Asia	499,148 (383,294, 618,553)	153.16 (120.38, 187.96)	837,440 (646,086, 1,048,392)	127.24 (99.25, 156.79)	−0.71 (−0.83 – −0.58)
Southern Latin America	54,264 (42,169, 67,119)	114.16 (89.03, 140.94)	84,879 (65,985, 106,072)	99.92 (78.01, 123.88)	−0.44 (−0.47 – −0.4)
Southern Sub-Saharan Africa	34,346 (26,635, 42,376)	108.23 (85.04, 132.63)	53,555 (41,101, 66,718)	91.8 (72.11, 113.84)	−0.55 (−0.65 – −0.44)
Tropical Latin America	52,039 (39,962, 64,760)	50.99 (40, 63.76)	101,293 (79,326, 127,258)	40.92 (32.09, 51.16)	−0.64 (−0.89 – −0.39)
Western Europe	381,063 (293,215, 477,398)	66.78 (51.81, 82.74)	542,416 (417,287, 695,713)	60.48 (46.84, 75.14)	−0.23 (−0.33 – −0.14)
Western Sub-Saharan Africa	248,114 (192,028, 303,612)	210.64 (167.56, 255.66)	454,555 (350,969, 560,877)	167.26 (131.55, 203.59)	−0.79 (−0.94 – −0.65)

ASPR, age-standardized prevalence rate; EAPC, estimated annual percentage changes; UI, uncertainty interval; CI, confidence interval; SDI, socio-demographic index.

**Figure 1:**
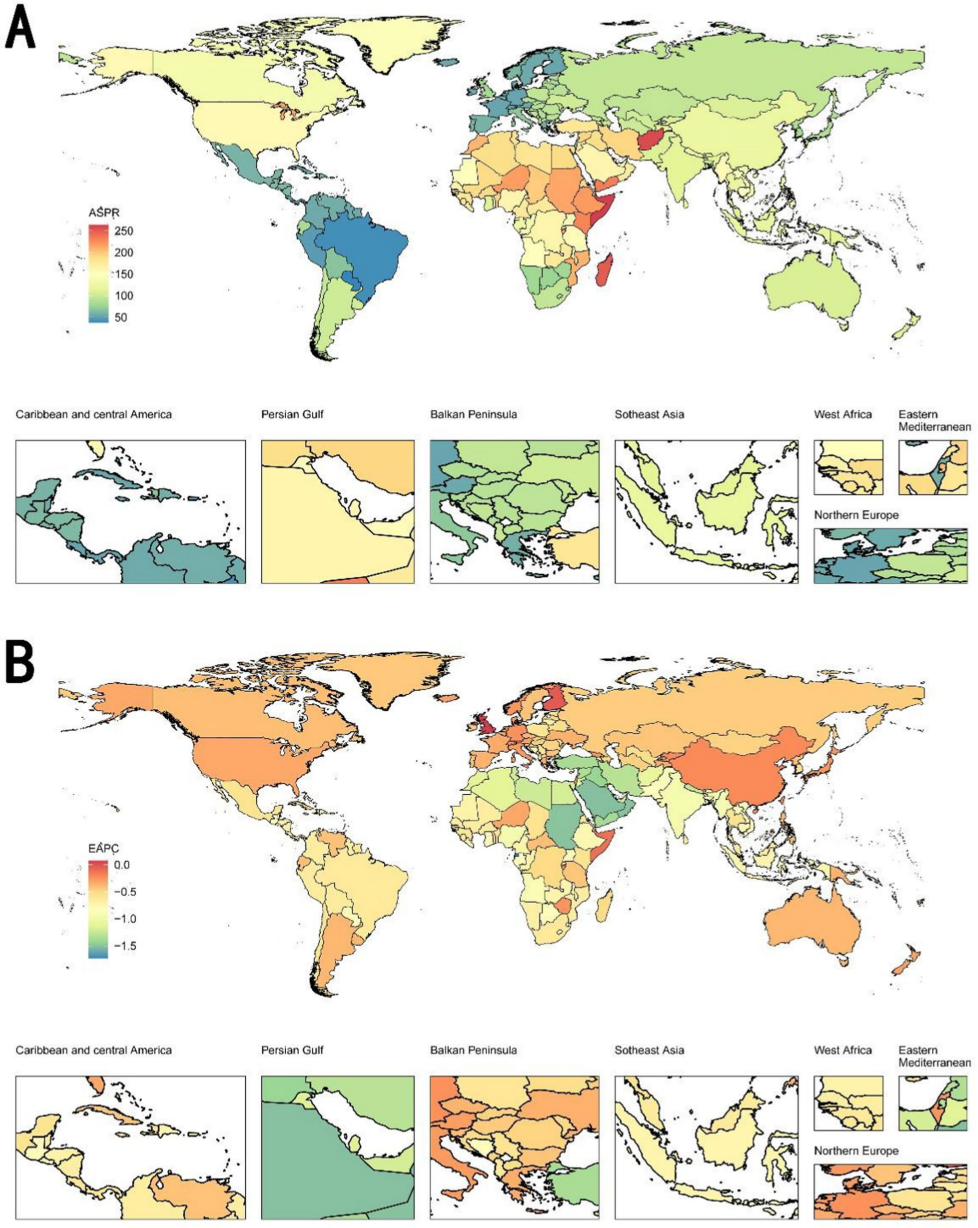
World maps of ASPR in 2021 and EAPC from 1992 to 2021 on complete hearing loss. **(A)** World map of ASPR in 2021. The Horn of Africa, parts of the Middle East, and specific regions in North Africa demonstrate notably higher ASPR values, while South America (particularly Brazil) and parts of Central America exhibit lower ASPR regions in South America, parts of Africa, and some countries in Eastern Europe show relatively higher ASPR. Conversely, many regions in North America, Northern Europe, and Southeast Asia exhibit lower ASPR values. **(B)** World map of EAPC from 1992 to 2021. Areas with slower reduction or slight increases in EAPC include North America, China, Australia, and parts of Northern Europe, while areas with higher reduction in EAPC include portions of the Middle East, Persian Gulf, and select African territories. ASPR, age-standardized prevalence rate; EAPC, estimated annual percentage change.

Supplementary Table S1 depicts the cases of prevalence and ASPR of complete hearing loss in 1992 and 2021, and its temporal trends from 1992–2021 among 204 countries and territories. In the world map of ASPR in 2021 (Figure 1A), the Horn of Africa, parts of the Middle East, and specific regions in North Africa demonstrate notably higher ASPR values, while South America (particularly Brazil) and parts of Central America exhibit lower ASPR values. In the world map of EAPC from 1992 to 2021 (Figure 1B), areas with slower reduction in EAPC include North America, China, Australia, and parts of Northern Europe, while areas with greater decreases in EAPC include portions of the Middle East, Persian Gulf, and select African territories.

### Prevalence rate in 1992 and 2021, and its relative change between 1992 and 2021

The prevalence rate increased by 16.13%, from 107.68 per 100,000 in 1992 to 125.05 per 100,000 in 2021 (Table 2). When examining gender differences, both males and females experienced an increase in prevalence rates, with females showing a slightly higher relative change of 16.81% compared to 15.25% in males (Table 2). High-SDI regions exhibited the most substantial increase in prevalence rate, rising by 31.80% from 138.8 per 100,000 in 1992 to 182.94 per 100,000 in 2021 (Table 2). Similarly, high-middle SDI regions saw a 35.46% increase in prevalence. In contrast, low SDI regions experienced a decrease in prevalence by 14.54%. Geographically, East Asia witnessed a dramatic increase of 62.30% in the prevalence rate of complete hearing loss, the highest among all regions (Table 2). High-income Asia Pacific also saw a notable increase of 83.97%. Conversely, regions like Central Sub-Saharan Africa and Eastern Sub-Saharan Africa experienced declines in prevalence rates by 15.50 and 8.72%, respectively. In Western Sub-Saharan Africa, the prevalence rate decreased significantly by 23.67%. However, these trends contrast with the increases seen in regions such as Central Europe, where the prevalence rate rose by 32.94%, and Central Latin America, which saw a significant increase of 41.10%.

**Table 2:** The prevalence rate of complete hearing loss in 1992 and 2021, and its relative change between 1992 and 2021.

Characteristics	Prevalence rate in 1992	Prevalence rate in 2021	1992–2021
			Relative change (%)
Global	107.68 (83.35, 132.34)	125.05 (98.28, 153.62)	16.13
Male	98.69 (75.66, 121.12)	113.74 (88.9, 140.46)	15.25
Female	116.81 (91.3, 142.81)	136.44 (107.34, 167.43)	16.81
Low SDI	113.97 (88.51, 138.82)	97.4 (75.04, 119.22)	−14.54
Low-middle SDI	99.02 (76.46, 121.69)	97.09 (75.09, 118.92)	−1.95
Middle SDI	93.8 (71.69, 115.57)	119.99 (93.12, 149.76)	27.92
High-middle SDI	111.43 (86.62, 136.86)	150.94 (117.8, 188.82)	35.46
High SDI	138.8 (109.01, 170.96)	182.94 (141.6, 227.95)	31.80
Andean Latin America	43.67 (33.4, 53.34)	54.45 (42.24, 67.09)	24.69
Australasia	136.89 (108.01, 167.77)	176.36 (135.13, 223.56)	28.83
Caribbean	49.91 (38.74, 60.94)	61.69 (48.15, 77.07)	23.60
Central Asia	80.98 (62.43, 100.39)	84.9 (64.87, 105.46)	4.84
Central Europe	120.52 (92.52, 150.19)	160.22 (123.21, 200.69)	32.94
Central Latin America	40.15 (30.82, 50.24)	56.65 (43.64, 70.42)	41.10
Central Sub-Saharan Africa	97.35 (75.16, 119.09)	82.26 (62.21, 102)	−15.50
East Asia	100.02 (76.08, 124.96)	162.33 (124.42, 205.7)	62.30
Eastern Europe	126.48 (96.58, 157.5)	149.75 (115.51, 185.96)	18.40
Eastern Sub-Saharan Africa	114.91 (89.89, 138.93)	104.89 (80.76, 127.67)	−8.72
High-income Asia Pacific	112.2 (86.44, 140.38)	206.41 (157.62, 264.6)	83.97
High-income North America	208.99 (163.73, 259.92)	248.43 (192.71, 313.24)	18.87
North Africa and Middle East	185.84 (144.61, 232.48)	159.67 (122.36, 195.36)	−14.08
Oceania	64.22 (48.51, 80.29)	66.19 (50.07, 83.22)	3.07
South Asia	89.95 (69.63, 110.35)	92 (71.2, 113.58)	2.28
Southeast Asia	103.62 (79.57, 128.41)	119.92 (92.52, 150.13)	15.73
Southern Latin America	106.87 (83.05, 132.18)	125.39 (97.47, 156.69)	17.33
Southern Sub-Saharan Africa	62.63 (48.57, 77.27)	66.69 (51.18, 83.08)	6.48
Tropical Latin America	33.02 (25.36, 41.1)	44.52 (34.86, 55.93)	34.83
Western Europe	98.39 (75.71, 123.26)	124.01 (95.4, 159.06)	26.04
Western Sub-Saharan Africa	121.57 (94.09, 148.77)	92.8 (71.65, 114.5)	−23.67

SDI, Socio-demographic Index.

**Figure 2:**
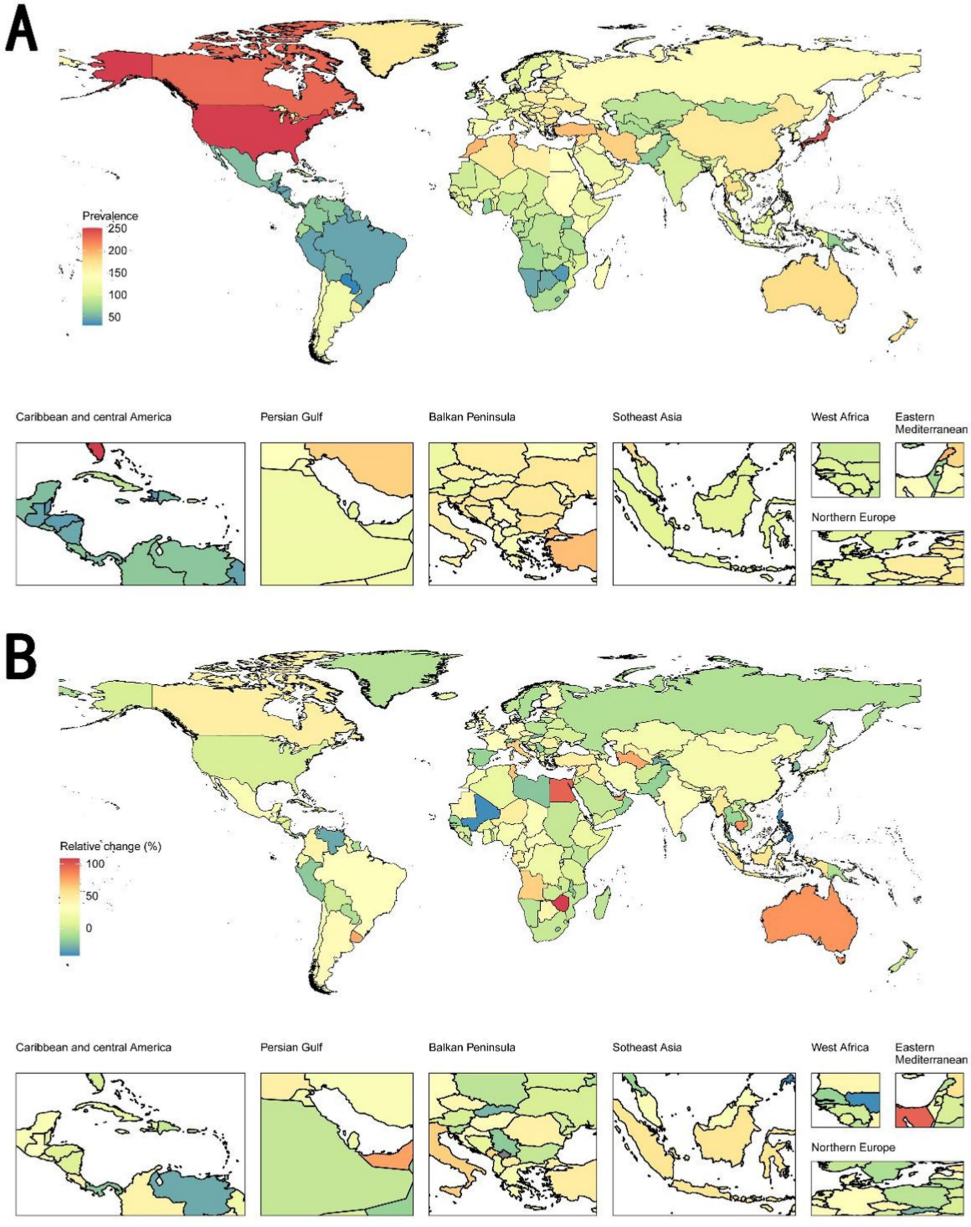
World maps of prevalence rate in 2021 and relative change from 1992 to 2021 on complete hearing loss. **(A)** World maps of prevalence rate in 2021. The United States and Canada exhibited the highest prevalence rates globally, while Brazil, Colombia, and Peru demonstrate substantially lower rates. **(B)** World map of relative change from 1992 to 2021. Regions marked in red indicate an increase in prevalence, while regions in green show a decrease. Sudan and Mauritania exhibit high increases of relative change, while Nigeria, Angola, and Algeria show notable decreases.

Supplementary Table S2 depicts the prevalence rate of complete hearing loss in 1992 and 2021, and its relative change from 1992–2021 among 204 countries and territories. In the world map of prevalence in 2021 (Figure 2A), the United States and Canada exhibited the highest prevalence rates globally, while Brazil, Colombia, and Peru demonstrate substantially lower rates. In the world map of relative change from 1992 to 2021 (Figure 2B), Sudan and Mauritania exhibit high increases of relative change, while Nigeria, Angola, and Algeria show notable decreases.

### Age and temporal trends on prevalence

**Figure 3:**
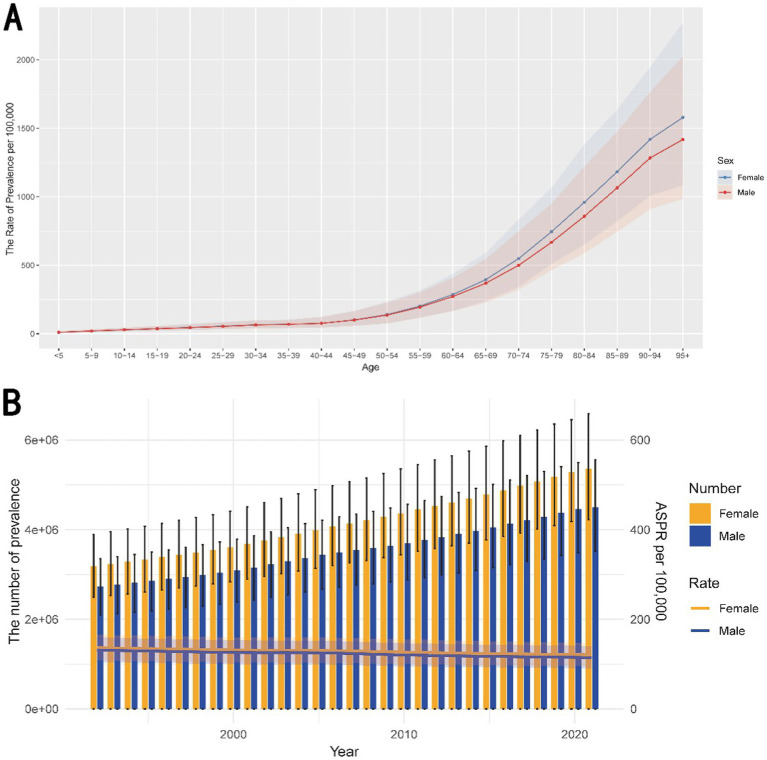
Age and temporal trends on prevalence of complete hearing loss. **(A)** The trend of prevalence with the increasing age. The graph illustrates the rate of prevalence per 100,000 population across different age groups in 2021, separately for males and females. The prevalence of hearing loss increases sharply with age, particularly after age of 60. This trend suggests that aging is a significant risk factor for hearing loss, with a higher burden observed in older females compared to males. **(B)** The temporal trend on prevalence from 1992 to 2021. It displays the temporal trends in the number of prevalent cases and ASPR per 100,000 population from 1992 to 2021, again disaggregated by sex. The number of prevalent cases has steadily increased over the years for both males and females, while ASPR has slightly decreased during the same period. ASPR, age-standardized prevalence rate.

Figure 3A illustrates that the prevalence of complete hearing loss increases sharply with age in 2021, particularly after age of 60. The rate is slightly higher in females compared to males, especially in the older age groups. Figure 3B displays that the number of prevalent cases has steadily increased over the years for both males and females, while ASPR has slightly decreased during the same period.

### The number of prevalence in different ages and SDI in 2021

Supplementary Figure S1A shows that the prevalence increases significantly with age, with a noticeable gender disparity. Females exhibit higher prevalence rates than males in most age groups, particularly from ages 45–90, where the difference is most pronounced. Supplementary Figure S1B illustrates that the prevalence is highest in middle SDI regions, with females showing a higher prevalence compared to males.

### Joinpoint regression analysis of ASPR

Table 3 and Supplementary Figure S2A shows that for males, the ASPR steadily decreased from 1992 to 2021, with three distinct phases identified by the joinpoint analysis. The 1992–2000 period showed significant decline (APC = −0.54%, *p* < 0.001), followed by a plateau phase during 2000–2005 (APC = −0.05%, *p* = 0.28). The most pronounced reduction occurred during 2005–2011 (APC = -0.82%, *p* < 0.001), followed by a moderate but significant continuing decline through 2011–2021 (APC = −0.44%, *p* < 0.001). Supplementary Figure S2B demonstrates that for females, the ASPR also experienced a continuous decline over the study period. The rate of decrease was slightly more gradual in females compared to males. The first segment from 1992 to 2000 showed a significant decline (APC = −0.47, *p* < 0.001), followed by a slower decline from 2000 to 2005 (APC = −0.14, *p* < 0.001). The decline became more pronounced from 2005 to 2015 (APC = −0.55, *p* < 0.001), and this trend persisted from 2015 to 2021 (APC = −0.35, *p* < 0.001).

**Table 3:** Joinpoint regression analysis: trends in ASPR among both sexes, males, and females, 1992–2021.

Gender	Period	APC (95% CI)	*p* value for APC	AAPC (95% CI)	*p* value for AAPC
Both	1992–2000	−0.51 (−0.52 – −0.49)	<0.001	−0.45 (−0.46 – −0.43)	<0.001
	2000–2005	−0.09 (−0.14 – −0.04)	0.002		
	2005–2009	−0.71 (−0.79 – −0.63)	<0.001		
	2009–2014	−0.57 (−0.62 – −0.52)	<0.001		
	2014–2021	−0.40 (−0.42 – −0.37)	<0.001		
Female	1992–2000	−0.47 (−0.49 – −0.45)	<0.001	−0.42 (−0.43 – −0.41)	<0.001
	2000–2005	−0.14 (−0.19 – −0.09)	<0.001		
	2005–2015	−0.55 (−0.57 – −0.54)	<0.001		
	2015–2021	−0.35 (−0.38 – −0.32)	<0.001		
Male	1992–2000	−0.54 (−0.57 – −0.51)	<0.001	−0.48 (−0.50 – −0.46)	<0.001
	2000–2005	−0.05 (−0.13–0.03)	0.28		
	2005–2011	−0.82 (−0.88 – −0.76)	<0.001		
	2011–2021	−0.44 (−0.46 – −0.42)	<0.001		

ASPR, age-standardized prevalence rate; APC, annual percent change; AAPC, average annual percent change.

### Age, period, and cohort effects on prevalence

For age, Supplementary Figure S3 shows a sharp increase in risk with advancing age. The relative risks (RRs) escalate significantly starting from the age group of 45–49 years, peaking in the oldest age group (90–94 years and 95+). For period, Supplementary Figure S3 shows a slight increase in relative risk across the periods from 1992–1996 to 2017–2021. For birth cohort, Supplementary Figure S3 shows a decreasing trend in relative risk across successive birth cohorts from 1897–1901 to 2017–2021. The detailed data is shown in Table 4.

**Table 4:** Age-Period-Cohort model analysis of prevalence.

Group	Variable	Coef	SE	*Z*	*p*	RR	95% CI	
							Lower	Upper
Age								
	<5	−1.820	0.001	−1569.070	<0.001	0.162	0.162	0.162
	5–9	−1.300	0.001	−1533.520	<0.001	0.272	0.272	0.273
	10–14	−1.028	0.001	−1410.440	<0.001	0.358	0.357	0.358
	15–19	−0.868	0.001	−1306.630	<0.001	0.420	0.419	0.420
	20–24	−0.757	0.001	−1219.490	<0.001	0.469	0.468	0.470
	25–29	−0.657	0.001	−1122.750	<0.001	0.518	0.518	0.519
	30–34	−0.567	0.001	−1012.540	<0.001	0.567	0.567	0.568
	35–39	−0.588	0.001	−1049.250	<0.001	0.555	0.555	0.556
	40–44	−0.570	0.001	−1023.610	<0.001	0.566	0.565	0.566
	45–49	−0.376	0.001	−716.880	<0.001	0.687	0.686	0.687
	50–54	−0.148	0.000	−299.700	<0.001	0.862	0.861	0.863
	55–59	0.117	0.000	251.290	<0.001	1.124	1.123	1.125
	60–64	0.379	0.000	855.020	<0.001	1.460	1.459	1.461
	65–69	0.610	0.000	1406.960	<0.001	1.840	1.838	1.841
	70–74	0.853	0.000	1960.940	<0.001	2.347	2.345	2.349
	75–79	1.072	0.000	2357.140	<0.001	2.922	2.919	2.925
	80–84	1.257	0.000	2514.730	<0.001	3.514	3.510	3.517
	85–89	1.389	0.001	2337.370	<0.001	4.010	4.005	4.015
	90–94	1.491	0.001	1865.370	<0.001	4.443	4.436	4.450
	95+	1.512	0.001	1129.080	<0.001	4.537	4.525	4.549
Period								
	1992 ~ 1996	−0.153	0.000	−558.430	<0.001	0.858	0.858	0.859
	1997 ~ 2001	−0.094	0.000	−366.430	<0.001	0.910	0.910	0.911
	2002 ~ 2006	−0.022	0.000	−89.750	<0.001	0.979	0.978	0.979
	2007 ~ 2011	0.033	0.000	140.870	<0.001	1.033	1.033	1.034
	2012 ~ 2016	0.087	0.000	375.460	<0.001	1.091	1.090	1.091
	2017 ~ 2021	0.149	0.000	626.180	<0.001	1.161	1.160	1.161
Birth cohort								
	1897–1901	1.005	0.004	224.450	<0.001	2.731	2.708	2.755
	1902–1906	0.910	0.002	442.950	<0.001	2.485	2.475	2.496
	1907–1911	0.822	0.001	656.910	<0.001	2.275	2.270	2.281
	1912–1916	0.742	0.001	794.320	<0.001	2.101	2.097	2.105
	1917–1921	0.677	0.001	861.980	<0.001	1.968	1.965	1.971
	1922–1926	0.583	0.001	856.620	<0.001	1.791	1.789	1.794
	1927–1931	0.497	0.001	795.870	<0.001	1.644	1.642	1.646
	1932–1936	0.418	0.001	707.440	<0.001	1.518	1.516	1.520
	1937–1941	0.335	0.001	587.280	<0.001	1.398	1.396	1.399
	1942–1946	0.261	0.001	461.590	<0.001	1.299	1.297	1.300
	1947–1951	0.183	0.001	325.270	<0.001	1.201	1.200	1.202
	1952–1956	0.104	0.001	185.060	<0.001	1.110	1.109	1.111
	1957–1961	0.021	0.001	36.140	<0.001	1.021	1.020	1.022
	1962–1966	−0.067	0.001	−113.200	<0.001	0.936	0.935	0.937
	1967–1971	−0.152	0.001	−255.650	<0.001	0.859	0.858	0.860
	1972–1976	−0.237	0.001	−390.530	<0.001	0.789	0.788	0.790
	1977–1981	−0.317	0.001	−512.850	<0.001	0.728	0.727	0.729
	1982–1986	−0.401	0.001	−649.870	<0.001	0.670	0.669	0.670
	1987–1991	−0.499	0.001	−803.530	<0.001	0.607	0.606	0.608
	1992–1996	−0.591	0.001	−887.820	<0.001	0.554	0.553	0.555
	1997–2001	−0.675	0.001	−880.240	<0.001	0.509	0.508	0.510
	2002–2006	−0.760	0.001	−841.580	<0.001	0.467	0.467	0.468
	2007–2011	−0.852	0.001	−767.870	<0.001	0.427	0.426	0.427
	2012–2016	−0.948	0.002	−628.960	<0.001	0.388	0.387	0.389
	2017–2021	−1.060	0.003	−383.220	<0.001	0.346	0.345	0.348

SE, standard error; RR, relative risk; CI, confidence interval.

Supplementary Figure S4 presents the Age-Period-Cohort analysis, showing the interaction between age, periods, and cohorts in determining the prevalence rates.

### Decomposition analysis of prevalence

Supplementary Figure S5A shows that population growth is the most significant contributor to the increase in complete hearing loss cases across all sexes, with the largest impact observed in females. In higher SDI regions, aging is the dominant factor driving the increase in prevalence of complete hearing loss (Supplementary Figure S5B). However, in lower SDI regions (low-middle and low SDI areas), population growth is the predominant factor.

#### Frontier analysis of ASPR

Supplementary Figure S6A demonstrates a positive correlation between SDI and the prevalence of complete hearing loss from 1992 to 2021. Higher SDI levels are associated with a decreased prevalence of hearing loss, with a noticeable upward trend over the two decades. Supplementary Figure S6B focuses on the trend analysis of specific countries.

#### Health inequality analysis of prevalence

In 1992, the SII was negative (−17.16) (Supplementary Figure S7A). However, by 2021, this trend had reversed, with an SII of 39.19, reflecting a higher prevalence of hearing loss in countries with higher SDI. Supplementary Figure S7B presents a concentration curve that compares the cumulative distribution of the population ranked by SDI with the cumulative fraction of hearing loss prevalence. The concentration index was 0.03 in 1992 and − 0.05 in 2021, indicating a shift toward a more equitable distribution.

### The correlation between SDI and ASPR of complete hearing loss

Supplementary Figure S8A suggests that regions with lower SDI, such as Sub-Saharan Africa and South Asia, have higher ASPR. These regions show a gradual decline in ASPR over time but remain higher compared to high SDI regions like Western Europe and North America. Supplementary Figure S8B indicates that countries having lower SDI, such as Somalia and Afghanistan, showing higher ASPR, while high SDI countries like Luxembourg and Singapore exhibit lower ASPR.

### Prevalence of complete hearing loss in different causes

**Figure 4:**
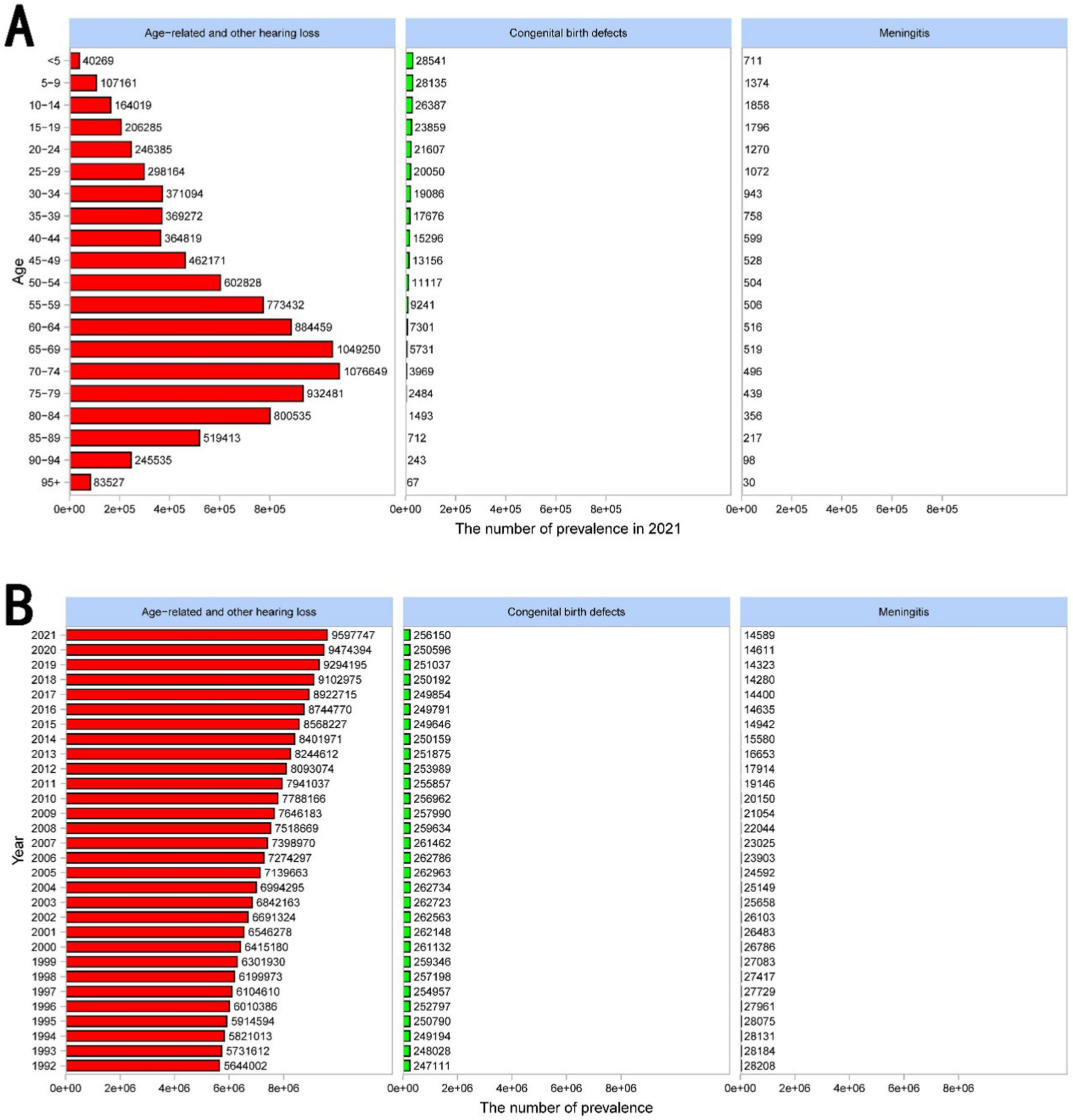
Prevalence of complete hearing loss across all age groups and years in different causes. **(A)** The bar chart displays the distribution of prevalence across various age groups in 2021. The prevalence of age-related and other hearing loss markedly increases with age, peaking in the 70–74 age group. This is followed by a slight decline in the older age groups. Congenital birth defects and hearing loss due to meningitis are significantly less prevalent and are relatively consistent across all age groups, with a noticeable concentration in younger populations for congenital birth defects. **(B)** The bar chart shows the temporal trends in the prevalence of these hearing loss types from 1992 to 2021. Age-related and other hearing loss has steadily increased over the years, reflecting the aging global population. Congenital birth defects and hearing loss due to meningitis, however, have shown only minor fluctuations over time, with congenital birth defects maintaining a slightly increasing trend, while the prevalence of hearing loss due to meningitis has a substantial decrease.

Figure 4A displays the distribution of prevalence across various age groups in 2021. The prevalence of age-related and other hearing loss markedly increases with age, peaking in the 70–74 age group. This is followed by a slight decline in the older age groups. Congenital birth defects and hearing loss due to meningitis are significantly less prevalent and are relatively consistent across all age groups, with a noticeable concentration in younger populations for congenital birth defects. Figure 4B shows the temporal trends in the prevalence of these hearing loss types from 1992 to 2021. Age-related and other hearing loss has steadily increased over the years, reflecting the aging global population. Congenital birth defects and hearing loss due to meningitis, however, have shown only minor fluctuations over time, with congenital birth defects maintaining a slightly increasing trend, while the prevalence of hearing loss due to meningitis has a substantial decrease.

Supplementary Figures S9–S11 illustrates the prevalence of age-related and other hearing loss, congenital birth defects, and meningitis across gender, SDI, and 21 regions in 2021.

These figures all indicate that age-related and other hearing loss is the dominant cause of complete hearing loss. According to the introduction of the GBD database, age-related and other hearing loss includes causes not identified as meningitis, chronic otitis media, or congenital, which is dominantly caused by presbycusis.

### Prediction of ASPR from 2022 to 2036

**Figure 5:**
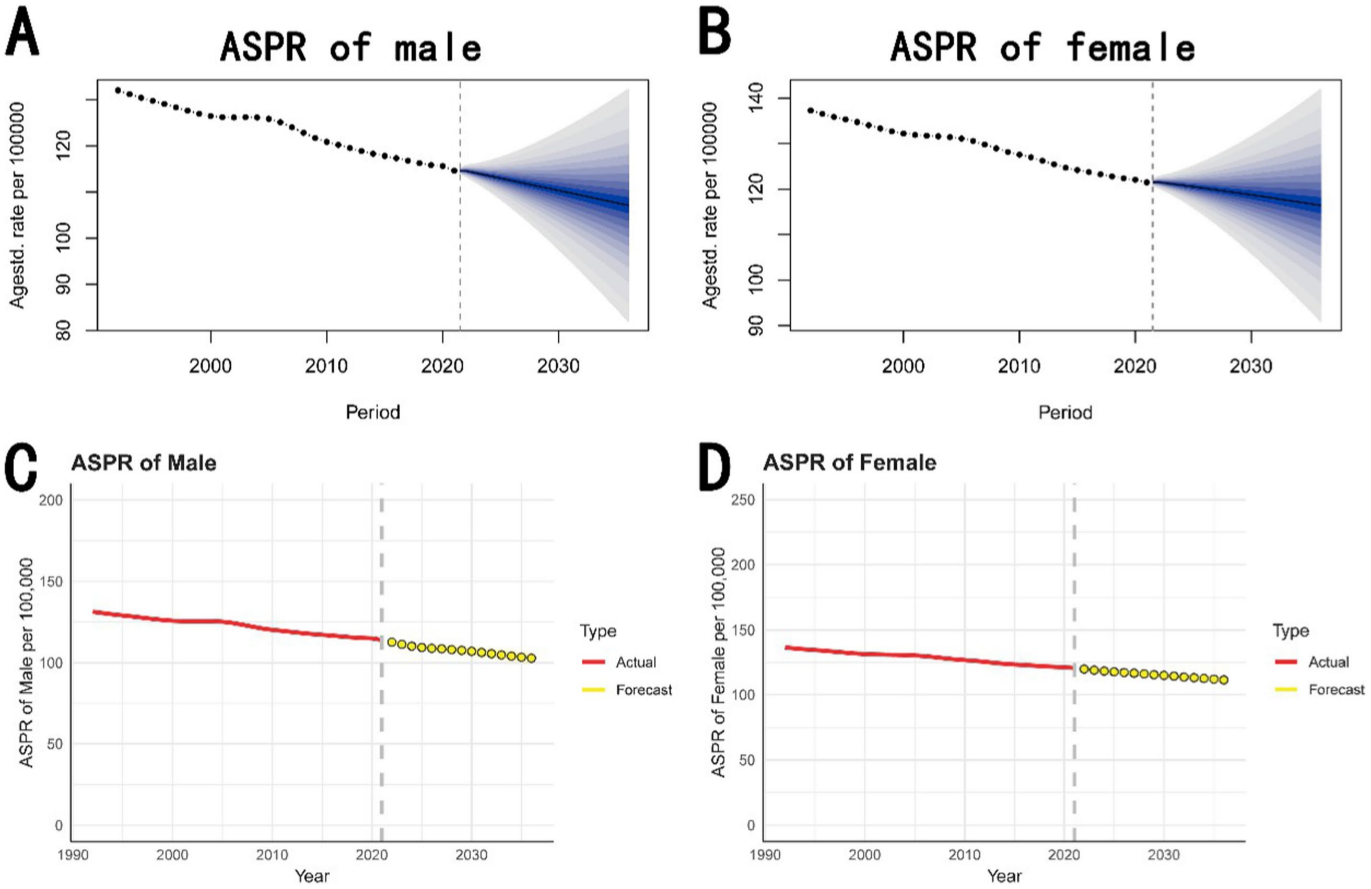
Prediction of ASPR from 2022 to 2036. **(A,B)** The prediction for males and females based on the BAPC model. It forecasts a decline in ASPR for both males and females from 2022 to 2036, with credible intervals indicating the uncertainty of these projections. **(C,D)** The prediction for males and females based on the ARIMA model. It similarly predicts a continuing decrease in ASPR, with forecasts extending to 2036. ASPR, age-standardized prevalence rate; BAPC, Bayesian age-period-cohort; ARIMA, autoregressive integrated moving average.

Figures 5A,B illustrates the BAPC model predictions for ASPR for both males and females from 2022 to 2036. The forecasted trend shows a potential stabilization in the decline, suggesting that the rate may plateau or slightly decrease further.

Figures 5C,D presents the ARIMA model forecasts for ASPR for both males and females from 2022 to 2036. It shows that while the prevalence of complete hearing loss may decrease over time, the reduction rate may slow down in the future.

## Discussion

This study provides a comprehensive analysis of the global trends in the prevalence and ASPR of complete hearing loss from 1992 to 2021, with a particular focus on age-related hearing loss. The findings indicate a global decline in ASPR, particularly among males, and significant regional and socio-demographic disparities. Regions with lower SDI levels, such as Sub-Saharan Africa and South Asia, experienced the most substantial reductions in ASPR, while high SDI regions, including East Asia, Western Europe, and North America, saw only modest declines. Despite the global decrease in ASPR, the overall prevalence of complete hearing loss has increased, particularly in high and high-middle SDI regions, driven largely by the aging population. The study underscores the growing public health challenge posed by age-related hearing loss, especially in regions with rapidly aging populations. The implications of both models (BAPC and ARIMA) showing a potential stabilization in the decline of ASPR for 2021–2036.

The impact of complete hearing loss on the quality of life in older adults is profound, affecting communication, social, emotional, and cognitive well-being (21, 22). Individuals with age-related hearing loss often struggle to maintain social connections and engage in everyday activities, leading to social isolation, depression, and anxiety (8, 23, 24). This isolation can exacerbate feelings of loneliness and contribute to mental health decline (25, 26). Age-related/untreated hearing loss increases the risk of developing dementia and other cognitive impairments (27–29).

Public health interventions aimed at reducing the burden of age-related hearing loss have shown varying degrees of effectiveness, depending on the socio-demographic context (3, 7, 30, 31). Age-related hearing loss (presbycusis) is the leading cause of hearing impairment in older adults, presenting a growing public health concern globally (21, 30). This condition, marked by a gradual decline in high-frequency hearing, worsens with age. As life expectancy increases, particularly in high-income regions, the prevalence of age-related hearing loss has risen significantly. Advanced healthcare systems in these areas have extended life spans, contributing to a higher prevalence of hearing loss as more people live longer with conditions that predispose them to auditory decline (31). High and high-middle SDI regions, including North America, Western Europe, and East Asia, have seen an increase in prevalence, driven largely by aging populations. These regions face a growing burden of age-related hearing loss, with East Asia experiencing a particularly dramatic rise. The advanced healthcare systems in these regions, while extending life spans, have also resulted in a higher prevalence of hearing loss among the older adult (32). High-SDI regions have more widespread in access to healthcare and hearing aids, with a modest decline in ASPR, suggesting positive impacts from these interventions (33). However, these improvements are not consistent across all low-SDI regions. Many still face substantial challenges, including limited resources, inadequate healthcare infrastructure, and barriers to healthcare access. Thus, the burden of hearing loss remains high, exacerbated by the lack of widespread public health interventions and limited availability of hearing aids and other assistive devices. It underscores the importance of early intervention and access to health interventions and hearing aids to mitigate cognitive decline and improve overall quality of life (34–36). To address these challenges, innovative approaches are needed, such as telemedicine, community-based education, and the development of affordable, low-cost hearing aids tailored for low-resource settings (37, 38). Public-private partnerships and international collaborations are crucial to making these technologies more accessible and affordable.

The trends of prevalence in complete hearing loss underscore the need for targeted public health strategies that address the specific challenges posed by complete hearing loss in aging populations. For example, in Western Europe and North America, where demographic shifts have led to an increase in the number of older adults at risk for hearing loss, studies suggest that up to 70% of individuals aged 70 and older may be impacted by this condition (18). Despite a modest global decline in ASPR, high-SDI regions have experienced less pronounced reductions, likely due to the overwhelming impact of aging populations. This highlights the need for public health strategies that specifically target the hearing health of older adults, including the development and implementation of age-specific hearing loss prevention programs and improved access to hearing aids and cochlear implants (39–41). People with presbycusis may benefit from novel therapies and treatments that would prevent or reverse hearing loss. In low to low-middle SDI regions, such as parts of Sub-Saharan Africa and South Asia, there has been a notable decline in the ASPR. This suggests that public health interventions, improved healthcare access, and educational programs aimed at hearing loss prevention are beginning to show positive effects. For example, Western Sub-Saharan Africa has made significant progress in reducing hearing loss over the past three decades, likely due to targeted initiatives and better access to basic healthcare services (42).

The stable decline of ASPR in for 2021–2036 may be contributed by expected improvements in hearing care accessibility and technological advancements in hearing aids and interventions (33, 38–40). However, the sustained number of prevalence may suffered from continued aging populations globally and limitations of current prevention strategies for age-related hearing loss in many countries (39–42).

This study offers several key strengths that enhance its contribution to the understanding of global hearing loss trends. Firstly, it utilizes comprehensive data from the GBD Study, covering an extensive timeframe from 1992 to 2021, allowing for a robust analysis of long-term trends and regional variations in complete hearing loss. The use of advanced statistical methods, including Joinpoint regression, Age-Period-Cohort modeling, and decomposition analysis, provides a nuanced understanding of the factors influencing hearing loss prevalence. Moreover, the study highlights age-related hearing loss (the most common form of hearing impairment among older adults), addressing a critical public health issue with growing global relevance. By highlighting the disparities between high and low-SDI regions, the research underscores the importance of targeted public health interventions and the need for equitable access to hearing care. Additionally, the projection of future trends up to 2036 offers valuable insights for policymakers and healthcare providers, aiding in the planning and implementation of effective hearing loss prevention and management strategies.

Several limitations need to be acknowledged. One limitation is the potential for reporting biases and inconsistencies in data collection across different regions, which could affect the accuracy of the findings. Additionally, the study did not account for potential confounding factors, such as variations in diagnostic criteria, access to healthcare services, and cultural differences, which could influence the observed trends in ASPR and prevalence. Another limitation is lack of the detailed cause of age-related and other hearing loss. Though age-related hearing loss is by far the largest cause of hearing loss, it is rarely recorded as a category in the GBD database.

## Conclusion

The prevalence of complete hearing loss remains high, particularly in aging populations within high-SDI regions, despite overall reductions in ASPR. Significant regional disparities remain, highlighting the need for targeted interventions to improve access to hearing care and affordable technologies in low-SDI regions. Despite progress in healthcare, significant disparities remain, underscoring the need for more equitable access to hearing care. Future research should prioritize understanding the diverse causes of hearing loss and developing affordable interventions for low-resource settings.

## Data Availability

Publicly available datasets were analyzed in this study. This data can be found here: http://ghdx.healthdata.org/gbd-results-tool.

## Footnotes

^1^ https://vizhub.healthdata.org/gbd-results/

^2^ https://ghdx.healthdata.org/data-type/estimate

^3^ https://surveillance.cancer.gov/joinpoint/download

^4^ https://www.r-project.org/
