# Supplementary material for: The global prevalence of complete hearing loss in 204 countries and territories from 1992 to 2021: a systematic analysis for the global burden of disease study 2021
Source: Front Public Health. 2025 Apr 9;13:1526719. doi: 10.3389/fpubh.2025.1526719 (PMC12039815; doi:10.3389/fpubh.2025.1526719)
Supplement: Supplementary file 1 [file Supplementary_file_1.docx]

**The global prevalence of complete hearing loss in 204 countries and territories from 1992 to 2021: a systematic analysis for the Global Burden of Disease Study 2021**

**Supplementary figures**

**Figure S1**: Prevalence of complete hearing loss by age and socio-demographic index (SDI) in 2021.

**Figure S2:** Joinpoint regression analysis of ASPR of complete hearing loss.

**Figure S3:** Age-period-cohort analysis on prevalence of complete hearing loss.

**Figure S4**: Detailed age-period-cohort (APC) analysis components.

**Figure S5**: Decomposition of prevalence by aging, epidemiological changes, and population growth.

**Figure S6**: Frontier analysis of ASPR.

**Figure S7**: Health inequality analysis of prevalence.

**Figure S8**: The correlation between SDI and ASPR of complete hearing loss.

**Figure S9**: Prevalence of complete hearing loss on gender in different causes in 2021.

**Figure S10**: Prevalence of complete hearing loss on SDI in different causes in 2021.

**Figure S11**: Prevalence of complete hearing loss 21 regions with different causes in 2021.

**Supplementary tables**

**Table S1** The cases of prevalence and ASPR of complete hearing loss in 1992 and 2021, and its temporal trends from 1992-2021 among 204 countries and territories.

**Table S2** The prevalence rate of complete hearing loss in 1992 and 2021, and its relative change from 1992-2021 among 204 countries and territories.


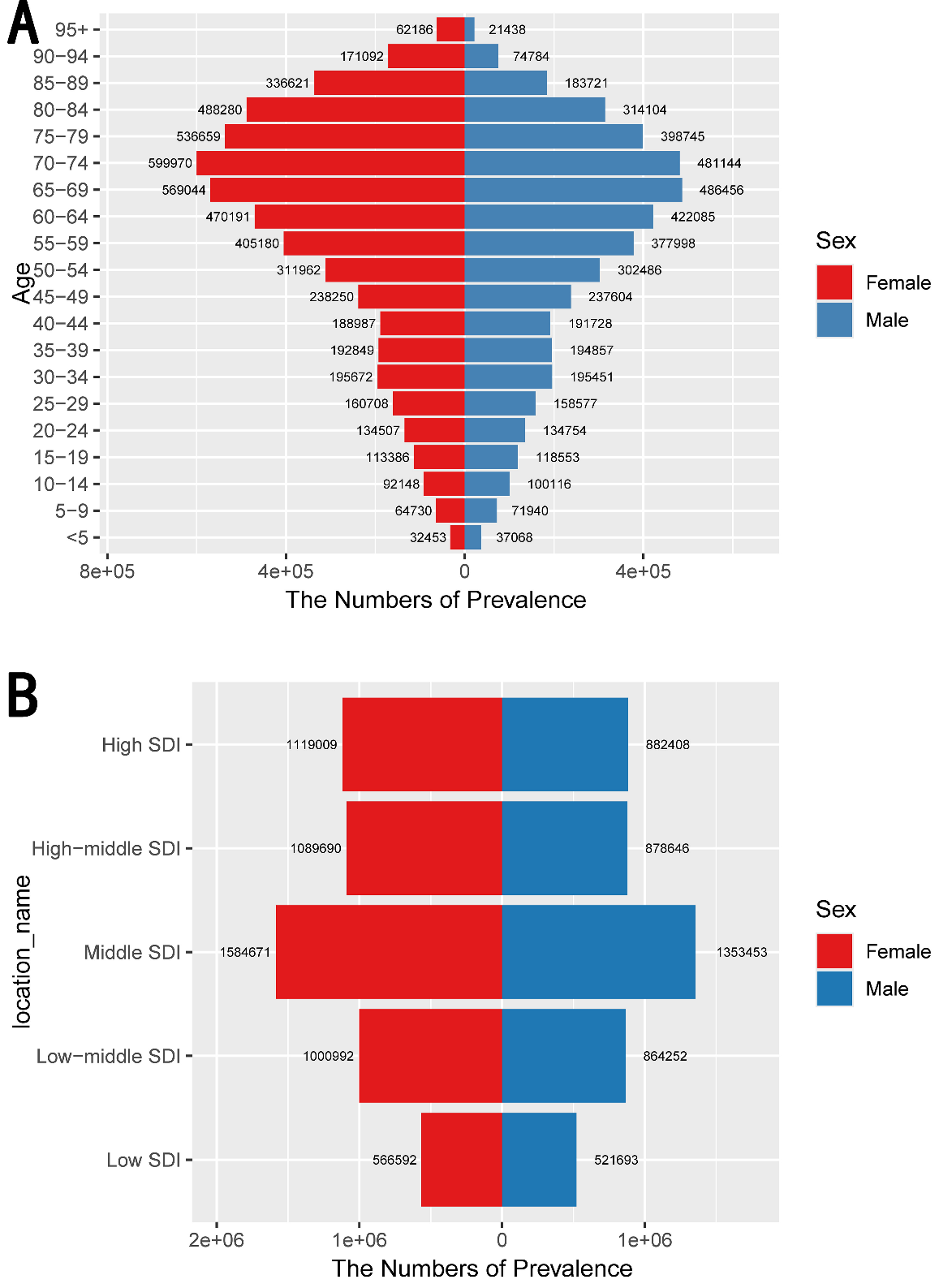


**Figure S1: Prevalence of complete hearing loss by age and socio-demographic index (SDI) in 2021.** (**A**) The number of prevalence in different age. The age pyramid shows the distribution of prevalence by age and sex in 2021, revealing higher prevalence in older age groups and a slightly higher burden among females. (**B**) The bar chart stratifies prevalence by SDI, indicating a higher burden in middle and low-middle SDI regions, with the lowest prevalence in high SDI regions.

SDI, socio-demographic index.
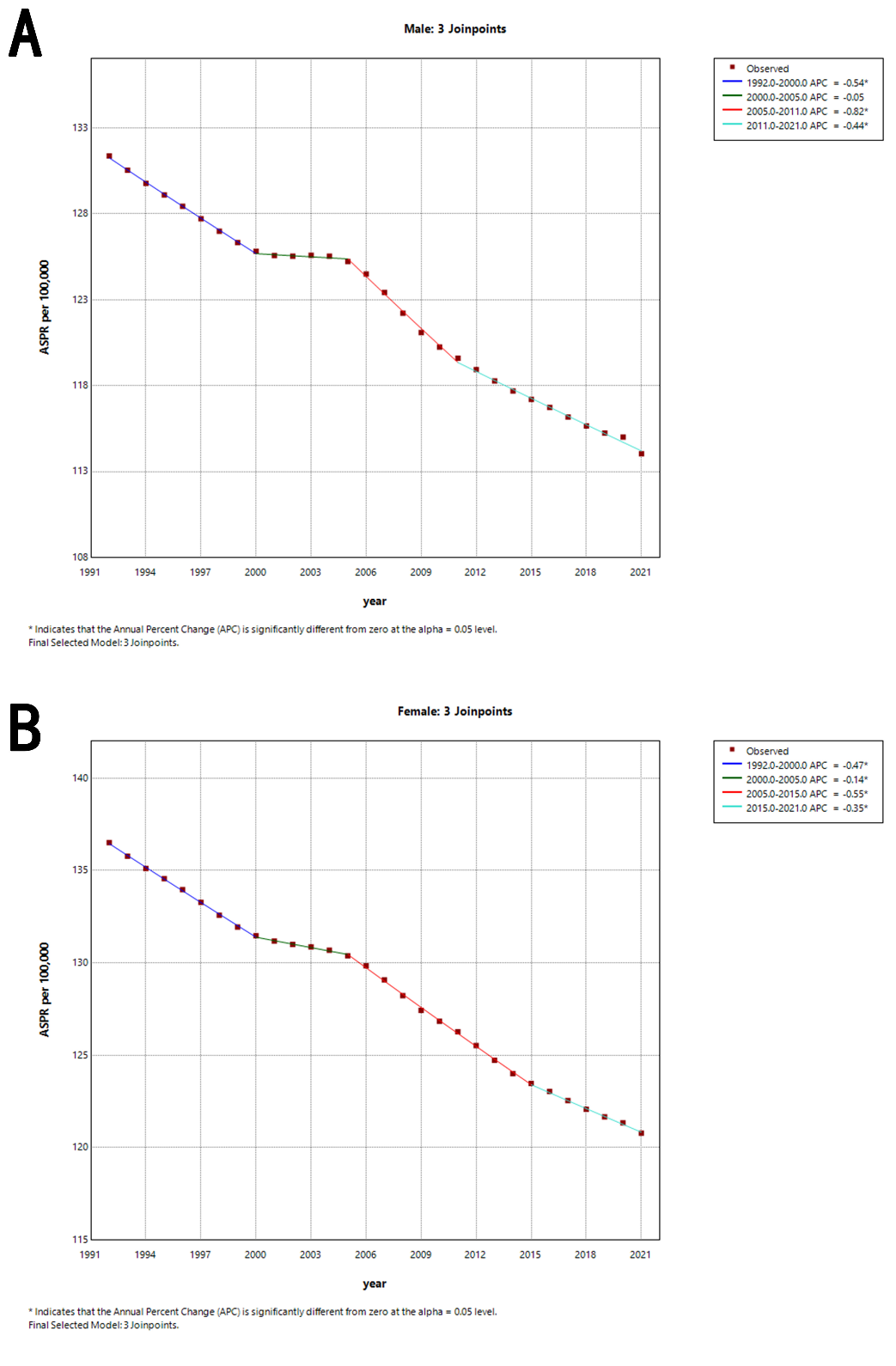


**Figure S2: Joinpoint regression analysis of ASPR of complete hearing loss.** (**A**) The joinpoint analysis for males. It indicates a significant decline in ASPR from 1992 to 2021, with three distinct periods of change. (**B**) The joinpoint analysis for females. It similarly shows a significant decrease in ASPR, with notable declines observed during the same periods. The Annual Percent Change (APC) is presented for each segment, indicating periods of significant change.

ASPR, age-standardized prevalence rate. APC, annual percent change.


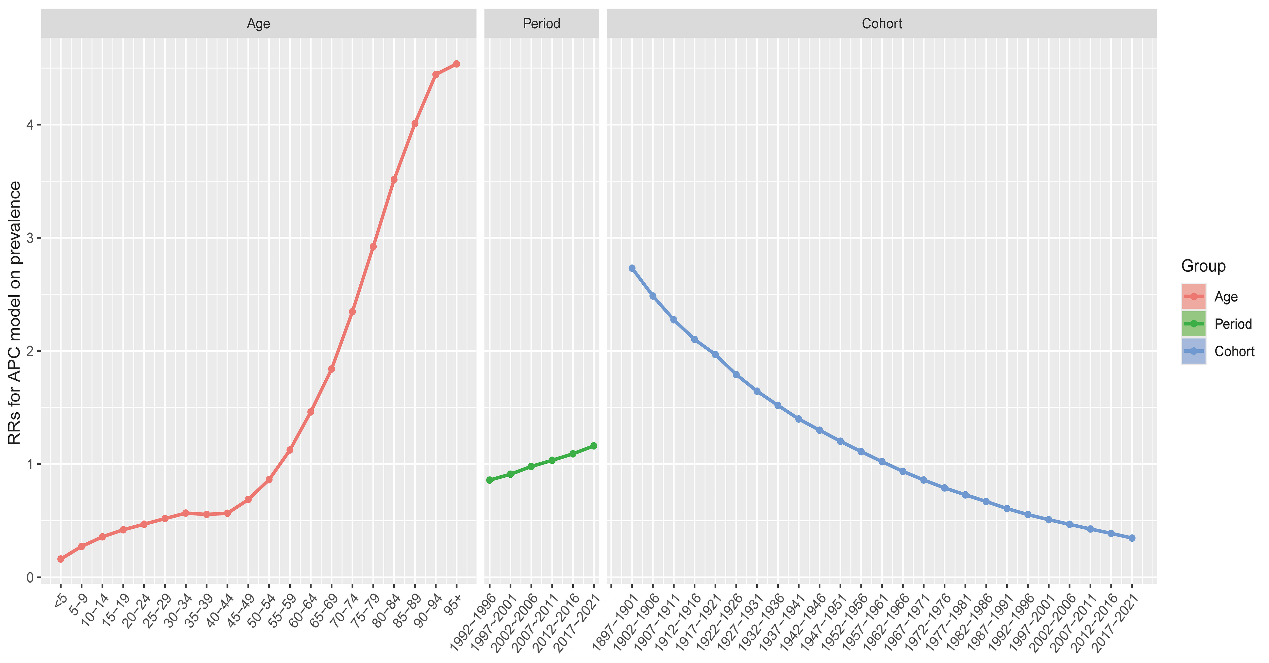


**Figure S3: Age-period-cohort analysis on prevalence of** **complete hearing loss.** The age-specific RRs show an increasing trend with age, especially after 50 years. The period-specific RRs show a modest increasing trend, suggesting a rise in risk over time. The cohort-specific RRs demonstrate a declining trend with more recent birth cohorts, indicating a generational reduction in risk.

RR, relative risk.


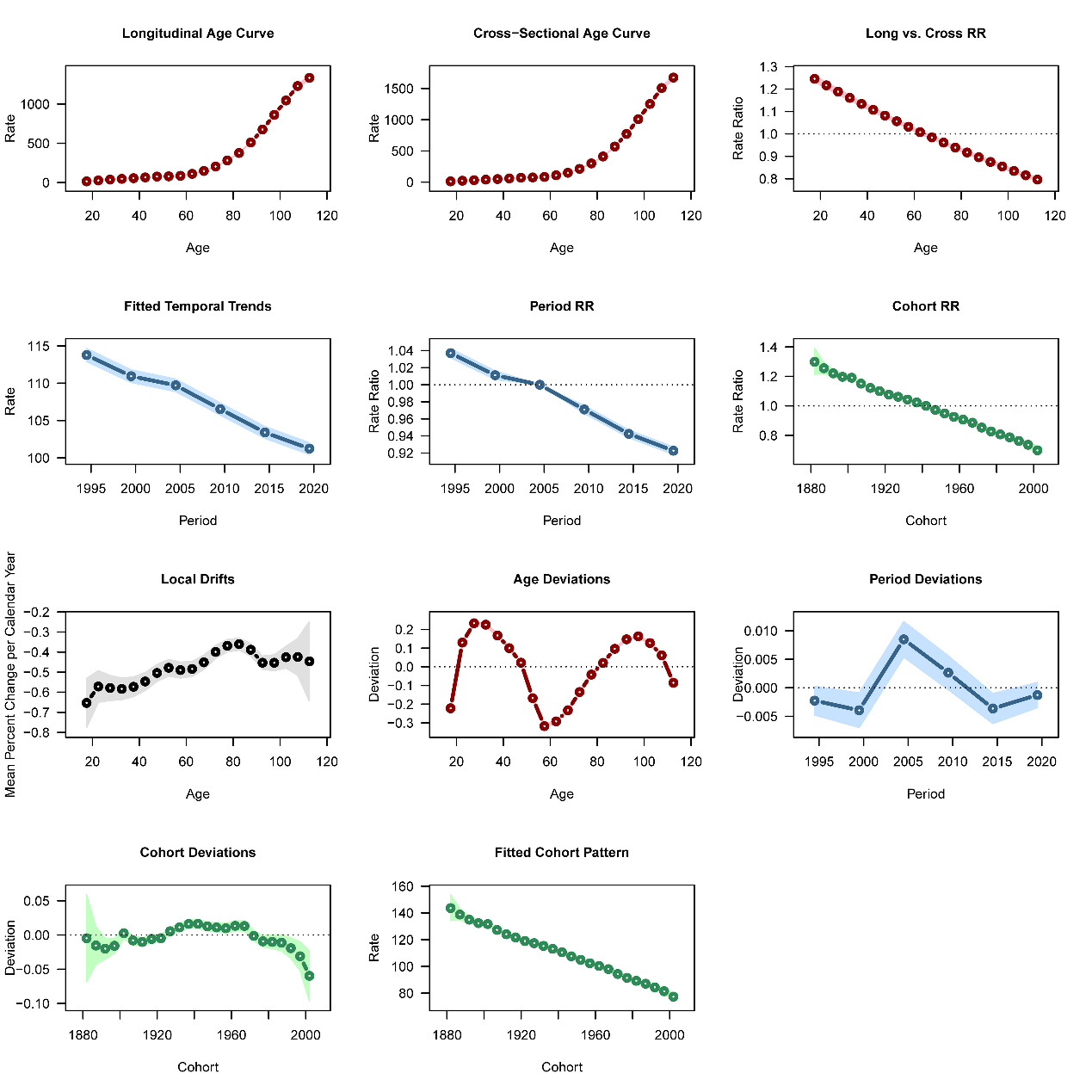


**Figure S4: Detailed age-period-cohort (APC) analysis components.** It presents the APC analysis, showing the interaction between age, periods, and cohorts in determining the prevalence rates. The Longitudinal and Cross-Sectional Age Curves demonstrate a sharp increase in hearing loss prevalence with age, particularly after age 60. Fitted Temporal Trends indicate a gradual decline in prevalence over time, while the Cohort RR suggests that more recent birth cohorts have a lower relative risk of hearing loss. Deviations in age, period, and cohort patterns illustrate the complex factors influencing hearing loss trends across different populations.

APC, age-period-cohort.


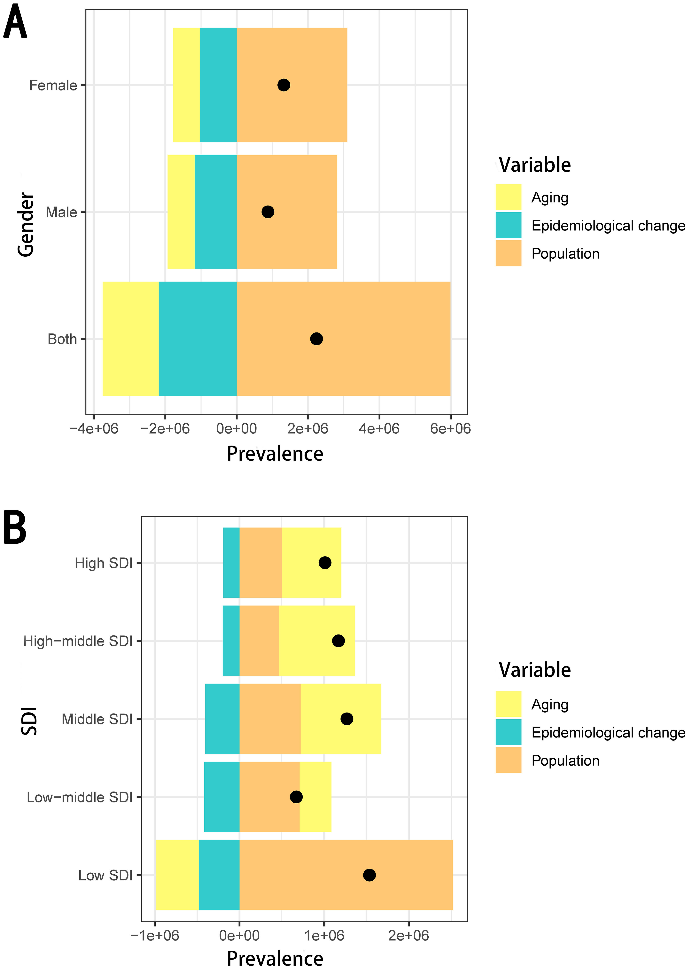


**Figure S5: Decomposition of prevalence by aging, epidemiological changes, and population growth.** (**A**) Decomposition analysis of prevalence by gender. It shows that population growth is the most significant contributor to the increase in complete hearing loss cases across all sexes, with the largest impact observed in females. Aging also plays a crucial role, particularly in females, but to a lesser extent than population growth. Epidemiological changes have a relatively minor impact and are more negative, indicating a slight improvement in hearing health over time. (**B**) Decomposition analysis of prevalence by SDI. It highlights the differences in contributing factors across SDI quintiles. In high SDI regions, aging is the dominant factor driving the increase in prevalence of complete hearing loss, followed by population growth. However, in lower SDI regions, particularly in low-middle and low SDI areas, population growth is the predominant factor, with aging and epidemiological changes contributing less significantly. The results suggest that demographic factors such as population growth and aging are major drivers of the increasing burden of hearing loss, with variations depending on socio-economic development.

SDI, socio-demographic index.


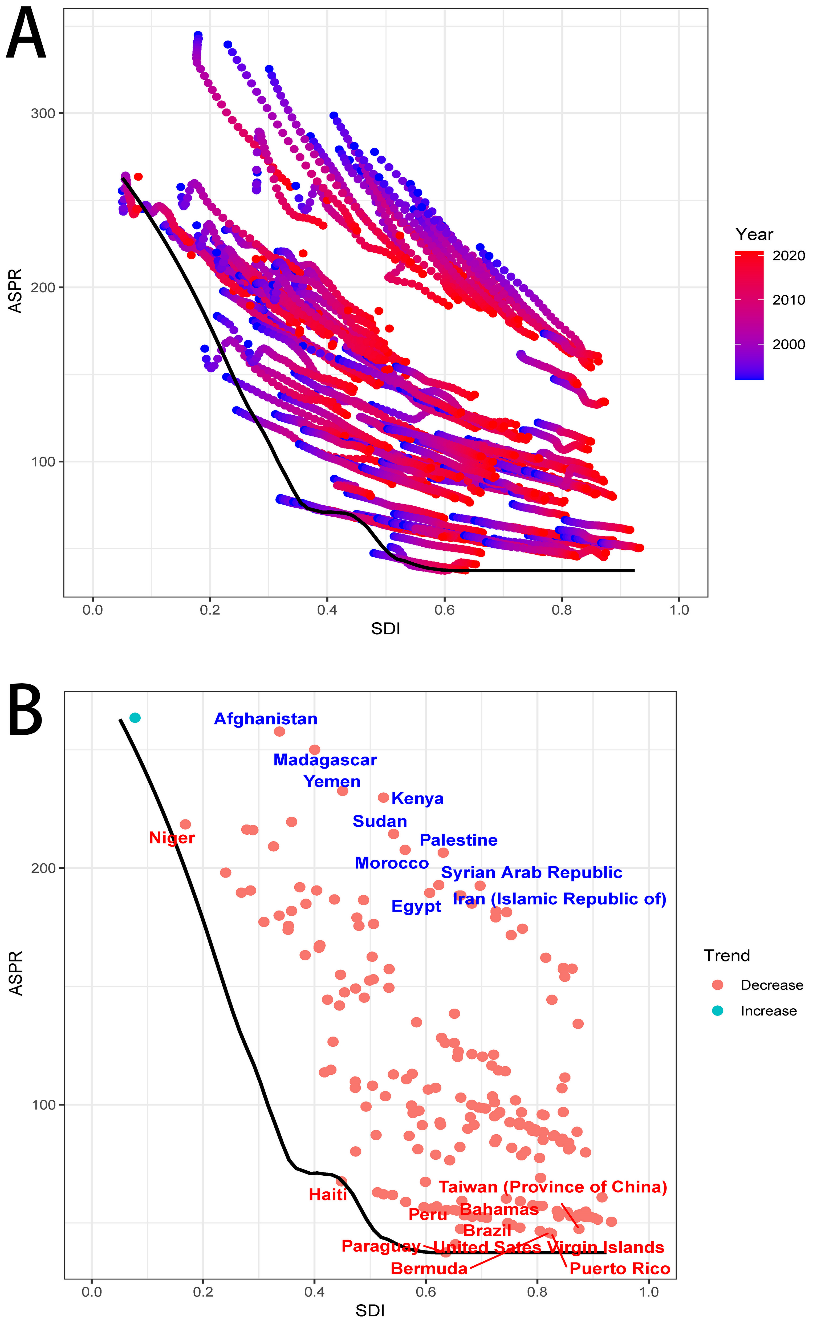


**Figure S6: Frontier analysis of ASPR.** (**A**) The scatter plot demonstrates a positive correlation between SDI and the prevalence of complete hearing loss from 1992 to 2021. Higher SDI levels are associated with a decreased prevalence of complete hearing loss, with a noticeable downward trend over the two decades. This trend is consistent across different years, showing that as countries develop socio-economically, the burden of complete hearing loss tends to decrease. (**B**) It focuses on the trend analysis of specific countries. It highlights the variation in the prevalence of hearing loss relative to SDI. Notably, the figure suggests that even within similar SDI levels, different countries can exhibit divergent trends in hearing loss prevalence, pointing to the influence of other factors such as healthcare access and public health interventions.

ASPR, age-standardized prevalence rate. SDI, socio-demographic index.


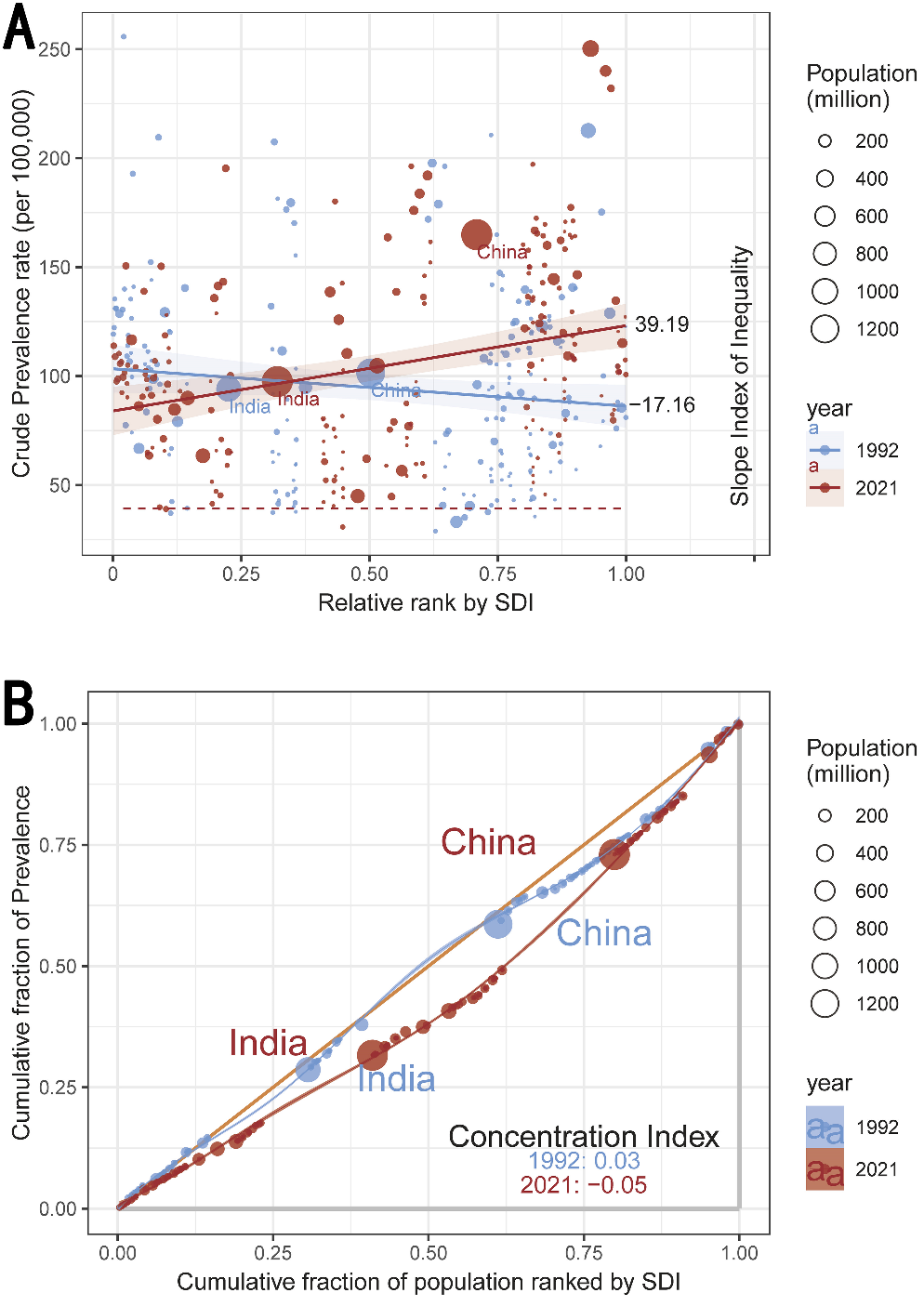


**Figure S7: Health inequality analysis of prevalence.** (**A**) It displays the relationship between crude prevalence rates of hearing loss and relative SDI ranks, using the Slope Index of Inequality (SII) as a measure. In 1992, the SII was negative (-17.16), indicating that lower SDI countries had higher prevalence rates of hearing loss. However, by 2021, this trend had reversed, with an SII of 39.19, reflecting a higher prevalence of hearing loss in countries with higher SDI. This shift suggests an increasing burden of hearing loss in more developed nations, possibly due to aging populations. (**B**) It presents a concentration curve that compares the cumulative distribution of the population ranked by SDI with the cumulative fraction of hearing loss prevalence. The concentration index, which quantifies the degree of inequality, was 0.03 in 1992, showing slight inequality in the distribution of hearing loss. By 2021, this index had decreased to -0.05, indicating a shift toward a more equitable distribution, albeit with a slight bias towards higher SDI countries. The larger circles representing China and India in both years highlight these countries' significant contributions to the global burden of hearing loss due to their large populations.

SDI, socio-demographic index. SII, slope index of inequality.


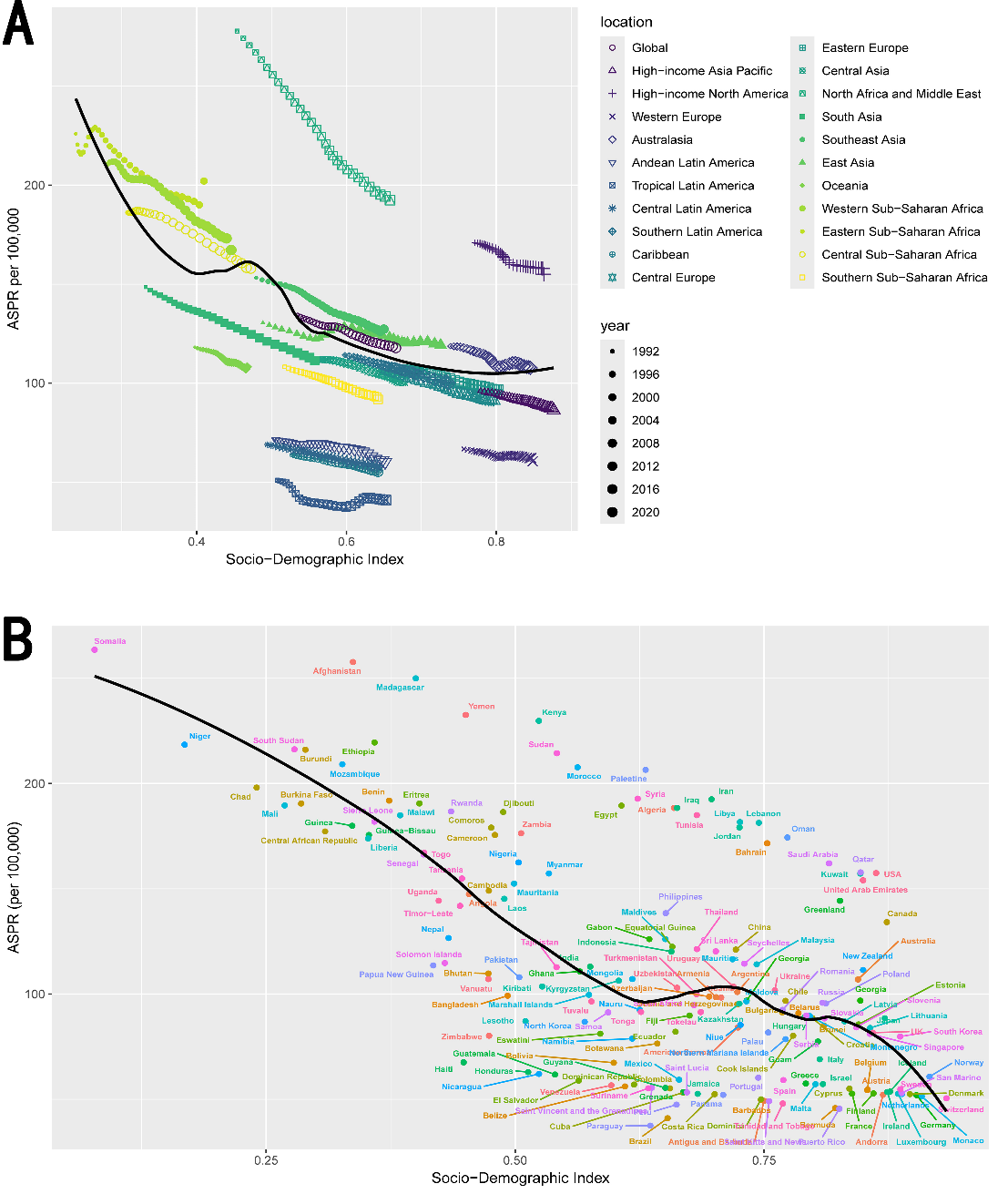


**Figure S8: The correlation between SDI and ASPR of complete hearing loss.** (**A**) The scatter plot illustrates the relationship between SDI and ASPR across 21 GBD regions from 1992 to 2021. It suggests that regions with lower SDI, such as Sub-Saharan Africa and South Asia, have higher ASPR. These regions show a gradual decline in ASPR over time but remain higher compared to high SDI regions like Western Europe and North America. (**B**) A more detailed view of the relationship is shown for individual countries, with labels indicating countries with notable trends relative to their SDI. It indicates that countries having lower SDI, such as Somalia and Afghanistan, show higher ASPR, while high SDI countries like Luxembourg and Singapore exhibit lower ASPR.

ASPR, age-standardized prevalence rate. SDI, socio-demographic index.


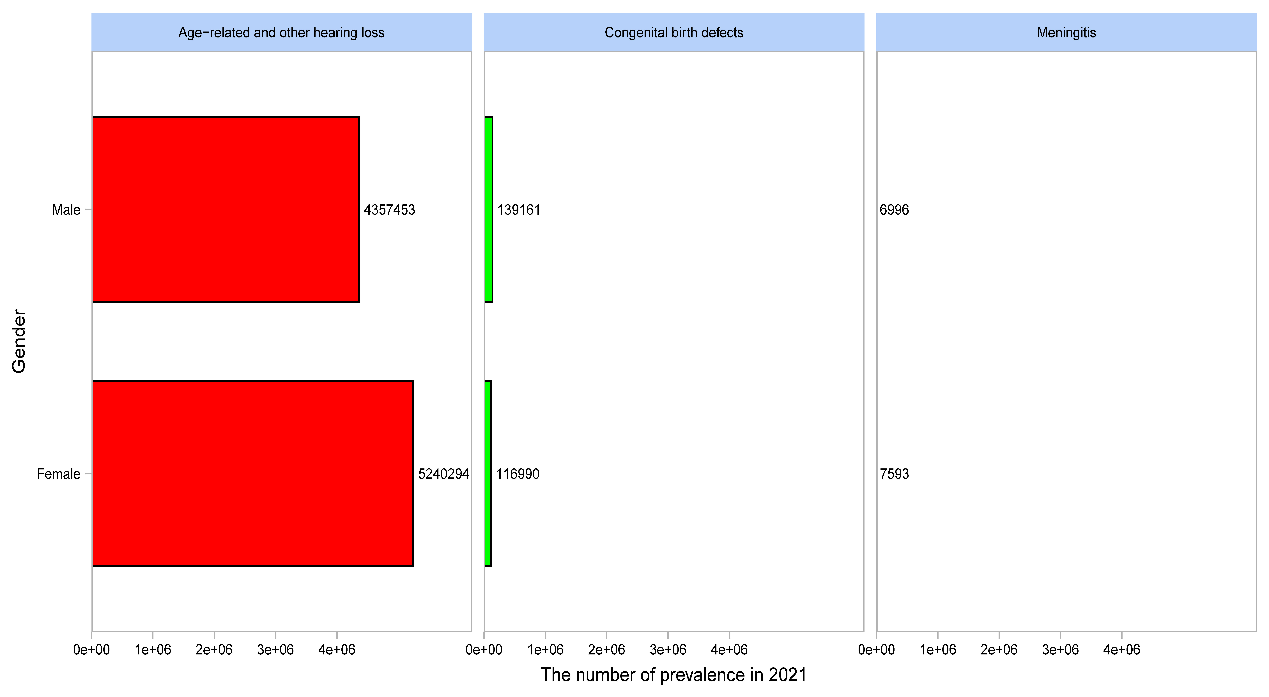


**Figure S9: Prevalence of complete hearing loss on gender in different causes in 2021.** The bar chart illustrates the gender-specific prevalence of age-related and other hearing loss, congenital birth defects, and meningitis in 2021. The data reveal significant disparities between males and females across these conditions. In terms of age-related and other hearing loss, females exhibit a higher prevalence, with a total of 5,240,294 cases, compared to 4,357,453 cases in males. This indicates that women are more affected by age-related hearing loss than men. However, for congenital birth defects and meningitis, the prevalence is higher in males. Males have 139,161 cases of congenital birth defects, compared to 116,990 cases in females. Similarly, for meningitis, males have 6,996 cases, while females have 7,593 cases. These findings underscore the importance of gender as a factor in the epidemiology of hearing loss and related conditions.


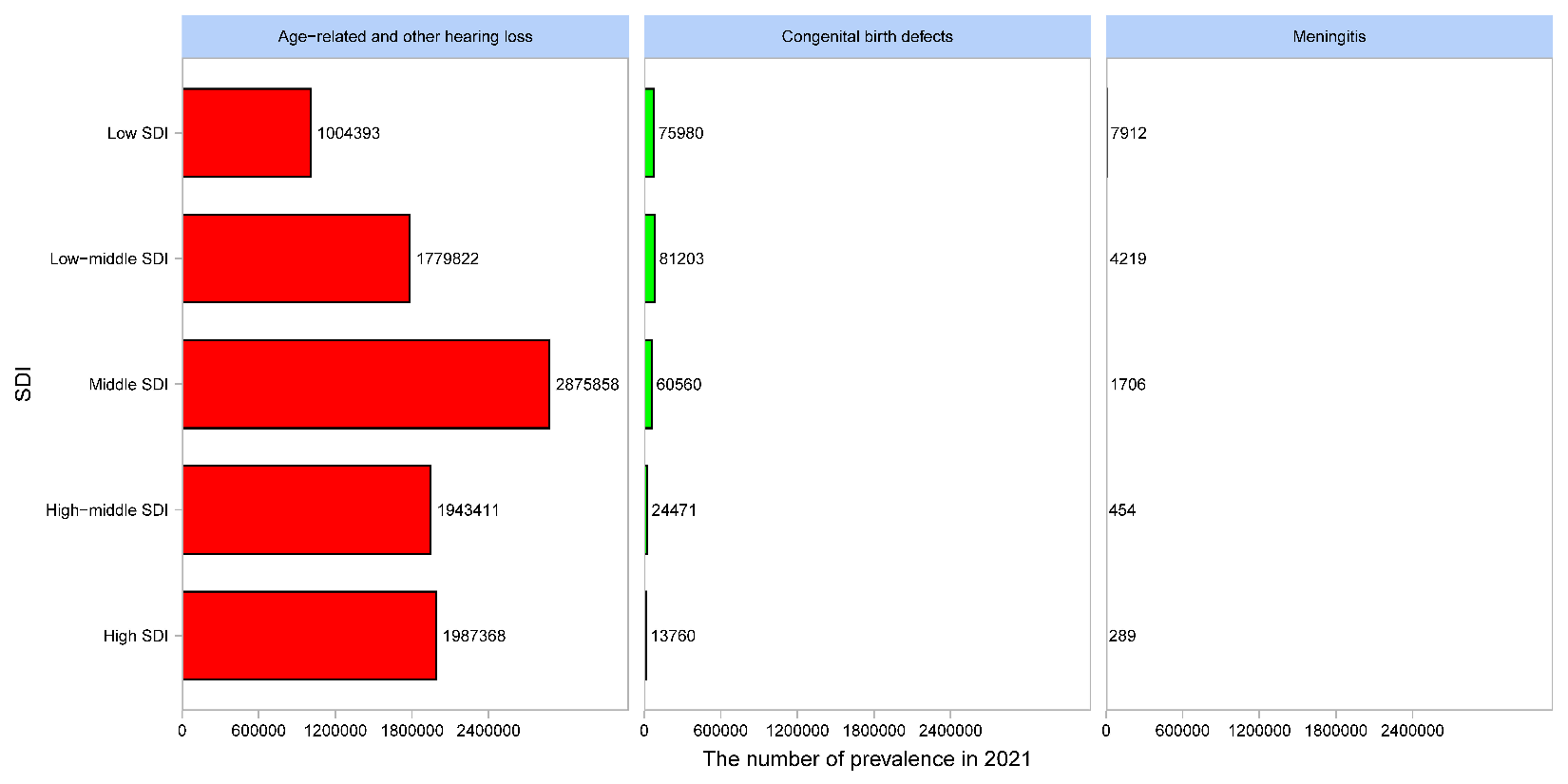


**Figure S10: Prevalence of complete hearing loss on SDI in different causes in 2021.**
The figure presents the prevalence of age-related hearing loss, congenital birth defects, and meningitis across five SDI levels in 2021. Age-related hearing loss and other hearing loss are most prevalent in middle-SDI regions, while congenital birth defects are most common in low-middle SDI regions. Meningitis shows the highest prevalence in low-SDI regions. These findings highlight disparities in health outcomes linked to socio-economic development levels.

SDI, socio-demographic index.


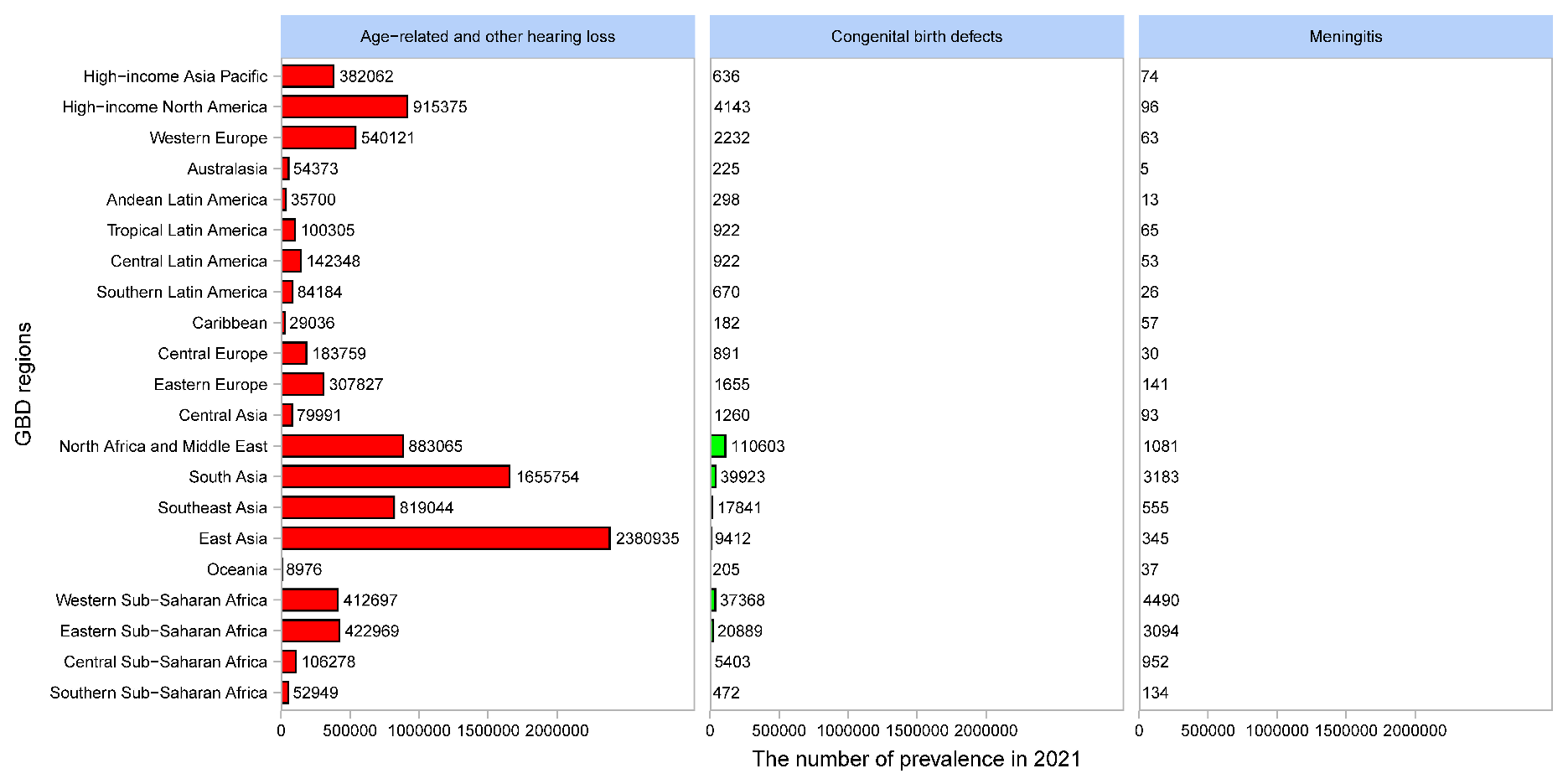


**Figure S11: Prevalence of complete hearing loss 21 regions with different causes in 2021.** It depicts the number of cases of age-related hearing loss, congenital birth defects, and meningitis across various GBD regions in 2021. East Asia, South Asia, and Eastern Europe are the most impacted by age-related hearing loss, while South Asia leads in congenital birth defects. Meningitis prevalence is highest in South Asia and Eastern Sub-Saharan Africa. These regional disparities emphasize the need for targeted public health strategies.

| **Table S1** The cases of prevalence and ASPR of complete hearing loss in 1992 and 2021, and its temporal trends from 1992-2021 among 204 countries and territories. | | | | | | |
| --- | --- | --- | --- | --- | --- | --- |
| **Characteristics** | **1992** | |  | **2021** | | **1992-2021** |
|  | **Cases of prevalence (95%UI)** | **ASPR per 100,000 (95%UI)** |  | **Cases of prevalence (95%UI)** | **ASPR per 100,000 (95%UI)** | **EAPC (95%CI)** |
| Afghanistan | 26471 (20745,32434) | 344.94 (272.43,418.47) |  | 47014 (36180,59771) | 257.75 (202.46,311.51) | -1.08 (-1.13 to -1.02) |
| Albania | 2690 (2041,3362) | 114.8 (89.44,141.22) |  | 3895 (2996,4906) | 98.35 (76.38,121.4) | -0.61 (-0.68 to -0.54) |
| Algeria | 45731 (35278,57036) | 259.2 (202.65,314.02) |  | 72329 (56140,88764) | 188.43 (148.48,229.07) | -1.13 (-1.18 to -1.09) |
| American Samoa | 26 (19,32) | 90.61 (70.53,111.61) |  | 38 (28,48) | 84.1 (64.57,103.27) | -0.3 (-0.43 to -0.17) |
| Andorra | 35 (27,44) | 55.68 (43.11,69.57) |  | 78 (60,99) | 52.19 (40.46,65.92) | -0.24 (-0.37 to -0.11) |
| Angola | 10384 (8024,12799) | 189.35 (150.37,230.06) |  | 23294 (17708,28867) | 147.47 (115.18,180.04) | -0.92 (-1.03 to -0.81) |
| Antigua and Barbuda | 34 (27,43) | 59.54 (46.04,73.97) |  | 49 (37,62) | 49.26 (38.4,62.34) | -0.65 (-0.75 to -0.56) |
| Argentina | 37289 (28894,46131) | 113.91 (88.47,140.48) |  | 55443 (42932,69208) | 100.99 (78.42,125.63) | -0.38 (-0.42 to -0.35) |
| Armenia | 3251 (2489,4068) | 115.1 (89.78,141.08) |  | 3992 (3074,5036) | 98.52 (76.86,122.93) | -0.62 (-0.69 to -0.55) |
| Australia | 23568 (18595,28829) | 117.98 (93.03,144.07) |  | 45755 (34913,58132) | 106.94 (82.58,134.53) | -0.37 (-0.54 to -0.21) |
| Austria | 6900 (5267,8842) | 59.38 (46.12,74.05) |  | 9665 (7416,12506) | 54.65 (42.17,68.67) | -0.31 (-0.43 to -0.18) |
| Azerbaijan | 6047 (4634,7532) | 110.62 (86.08,136.36) |  | 9599 (7208,11989) | 98.8 (76.12,121.64) | -0.5 (-0.58 to -0.42) |
| Bahamas | 95 (73,119) | 54.11 (41.9,67.35) |  | 177 (135,224) | 46.61 (35.82,59.1) | -0.55 (-0.64 to -0.46) |
| Bahrain | 784 (591,998) | 235.64 (184.93,286.73) |  | 2084 (1551,2666) | 171.67 (134.17,207.86) | -1.17 (-1.22 to -1.12) |
| Bangladesh | 75910 (59063,93331) | 129.49 (102.18,158.49) |  | 139391 (107655,171013) | 99.19 (78.15,121.71) | -1.02 (-1.18 to -0.85) |
| Barbados | 170 (130,217) | 55.74 (43.23,69.24) |  | 238 (182,306) | 49.74 (38.82,62.63) | -0.39 (-0.48 to -0.29) |
| Belarus | 13020 (10047,16244) | 104.54 (81.45,129.66) |  | 13317 (10242,16788) | 90.91 (70.49,112.93) | -0.54 (-0.61 to -0.47) |
| Belgium | 9052 (6883,11547) | 60.02 (46.51,75.05) |  | 12395 (9510,16010) | 54.4 (42.1,68.47) | -0.35 (-0.48 to -0.22) |
| Belize | 79 (61,96) | 68.5 (53.48,84.8) |  | 174 (134,213) | 56.13 (42.78,69.44) | -0.67 (-0.76 to -0.58) |
| Benin | 6565 (5178,8038) | 233 (184.96,282.06) |  | 14058 (10949,17357) | 191.91 (153.01,233.96) | -0.59 (-0.74 to -0.45) |
| Bermuda | 34 (26,43) | 54 (42.11,67.69) |  | 59 (44,75) | 45.85 (35.35,57.81) | -0.59 (-0.68 to -0.51) |
| Bhutan | 477 (367,595) | 148.68 (117.08,182.04) |  | 703 (549,864) | 109.78 (86.03,134.8) | -1.07 (-1.21 to -0.92) |
| Bolivia (Plurinational State of) | 3060 (2324,3784) | 82 (64.14,100.2) |  | 6163 (4683,7668) | 67.36 (52.14,83.59) | -0.64 (-0.69 to -0.58) |
| Bosnia and Herzegovina | 4952 (3827,6175) | 119.38 (94.08,147.21) |  | 5294 (4107,6677) | 95.09 (74.38,117.29) | -0.83 (-0.91 to -0.74) |
| Botswana | 660 (503,825) | 98.43 (76.47,122.98) |  | 1160 (872,1462) | 76.49 (59.07,95.57) | -0.82 (-0.97 to -0.67) |
| Brazil | 50802 (38996,63253) | 51.09 (40.07,63.9) |  | 99086 (77609,124413) | 41.02 (32.16,51.25) | -0.64 (-0.89 to -0.39) |
| Brunei Darussalam | 131 (99,163) | 97.38 (75.9,120.67) |  | 292 (219,365) | 85.05 (65.59,105.83) | -0.46 (-0.61 to -0.31) |
| Bulgaria | 11469 (8652,14506) | 102.45 (79.45,126.6) |  | 11702 (8834,14879) | 91.37 (70.42,114.06) | -0.44 (-0.51 to -0.37) |
| Burkina Faso | 13108 (10204,15990) | 232.46 (185.22,283.58) |  | 24063 (18426,29627) | 190.54 (150.09,234.2) | -0.65 (-0.78 to -0.51) |
| Burundi | 6627 (5201,8053) | 218.43 (171.93,263.89) |  | 14082 (10876,17207) | 216.09 (171.38,263.65) | -0.26 (-0.43 to -0.08) |
| Cabo Verde | 536 (431,657) | 198.38 (158.65,243.03) |  | 720 (558,886) | 149.43 (116.8,183.36) | -0.94 (-1.08 to -0.81) |
| Cambodia | 11401 (8842,14220) | 181.53 (142.12,222.38) |  | 20224 (15363,25264) | 149.09 (116.05,185.71) | -0.76 (-0.89 to -0.64) |
| Cameroon | 13442 (10385,16460) | 220.55 (173.1,267.63) |  | 30587 (23514,38057) | 175.6 (135.9,215.48) | -0.73 (-0.79 to -0.66) |
| Canada | 49133 (38228,61531) | 147.53 (114.67,185.19) |  | 86929 (66680,110427) | 134.18 (103.88,167.99) | -0.39 (-0.69 to -0.08) |
| Central African Republic | 2959 (2263,3632) | 197.79 (156.88,241.78) |  | 5067 (3839,6310) | 177.25 (139.15,215.46) | -0.41 (-0.51 to -0.3) |
| Chad | 8671 (6781,10596) | 234.94 (188.19,285.6) |  | 17308 (13406,21394) | 198.09 (158.24,243.14) | -0.55 (-0.68 to -0.41) |
| Chile | 12533 (9694,15564) | 114.37 (89.45,141.89) |  | 23929 (18539,29880) | 96.78 (75.67,119.59) | -0.57 (-0.61 to -0.53) |
| China | 1223765 (930175,1529094) | 132.51 (104.34,163.24) |  | 2345382 (1797292,2973656) | 121.13 (95.31,151.46) | -0.22 (-0.29 to -0.14) |
| Colombia | 13815 (10695,17250) | 66.41 (51.9,82.46) |  | 30440 (23716,38127) | 55.23 (42.8,69.57) | -0.66 (-0.75 to -0.57) |
| Comoros | 516 (401,628) | 201.02 (159.48,243.13) |  | 953 (743,1175) | 179.07 (140.68,222.06) | -0.59 (-0.76 to -0.42) |
| Congo | 2272 (1760,2799) | 165.04 (129.68,201.84) |  | 4338 (3295,5381) | 134.83 (106.25,167.22) | -0.71 (-0.82 to -0.6) |
| Cook Islands | 12 (9,15) | 90.59 (70.48,111.88) |  | 19 (15,24) | 80.24 (62.45,99.36) | -0.46 (-0.59 to -0.32) |
| Costa Rica | 1315 (1014,1629) | 62.39 (48.33,77.09) |  | 2848 (2200,3576) | 52.46 (40.39,65.75) | -0.6 (-0.69 to -0.51) |
| Croatia | 5754 (4412,7210) | 101.01 (79.1,124.63) |  | 7139 (5418,8974) | 88.64 (68.19,109.88) | -0.51 (-0.58 to -0.44) |
| Cuba | 6503 (4962,8065) | 60.22 (46.44,74.76) |  | 9855 (7574,12565) | 53.21 (41.49,66.57) | -0.44 (-0.53 to -0.35) |
| Cyprus | 517 (388,659) | 64.52 (50.38,81.15) |  | 1085 (832,1391) | 55.08 (42.97,69.41) | -0.54 (-0.67 to -0.41) |
| Czechia | 12613 (9595,15820) | 96.99 (75.37,119.84) |  | 16824 (12887,21552) | 86.7 (67.21,108.09) | -0.36 (-0.44 to -0.29) |
| C么te d'Ivoire | 12969 (9932,15999) | 201.95 (161.05,246.03) |  | 25397 (19409,31688) | 165.06 (131.97,205.56) | -0.6 (-0.74 to -0.46) |
| Democratic People's Republic of Korea | 15589 (11938,19498) | 95.97 (75.3,118.83) |  | 26467 (20398,32974) | 86.82 (68.13,107.13) | -0.4 (-0.58 to -0.22) |
| Democratic Republic of the Congo | 39539 (30591,48193) | 186.82 (149.36,227.3) |  | 77559 (58779,96321) | 163.19 (127.75,198.56) | -0.52 (-0.65 to -0.4) |
| Denmark | 4551 (3471,5803) | 56.86 (43.84,70.96) |  | 5961 (4534,7661) | 52.48 (40.43,65.79) | -0.29 (-0.41 to -0.16) |
| Djibouti | 474 (364,583) | 207.21 (163.61,252.56) |  | 1420 (1091,1761) | 186.5 (146.64,227.71) | -0.56 (-0.73 to -0.38) |
| Dominica | 38 (29,47) | 61.13 (47.5,75.85) |  | 39 (30,50) | 50 (39.13,62.7) | -0.68 (-0.77 to -0.59) |
| Dominican Republic | 3120 (2395,3863) | 69.95 (54.85,85.91) |  | 5747 (4500,7135) | 57.03 (44.66,71) | -0.73 (-0.82 to -0.64) |
| Ecuador | 6357 (4939,7765) | 98.33 (77.42,120.03) |  | 13627 (10405,16637) | 82.12 (63.02,101.23) | -0.41 (-0.53 to -0.28) |
| Egypt | 103284 (79826,128946) | 273.09 (215.2,334.54) |  | 146402 (112171,185458) | 189.54 (148.65,233.95) | -1.19 (-1.26 to -1.12) |
| El Salvador | 2552 (1969,3137) | 72.91 (56.39,89.06) |  | 3810 (2942,4718) | 58.88 (45.42,73.94) | -0.76 (-0.85 to -0.67) |
| Equatorial Guinea | 474 (364,581) | 193.44 (152.81,235.25) |  | 891 (673,1129) | 122.43 (94.86,150.21) | -1.73 (-1.84 to -1.62) |
| Eritrea | 3144 (2435,3922) | 213.36 (169.26,260.03) |  | 6640 (5125,8232) | 190.56 (151.14,231.95) | -0.58 (-0.76 to -0.41) |
| Estonia | 1935 (1500,2398) | 100.34 (78.03,122.87) |  | 2144 (1634,2730) | 85.62 (66.01,105.89) | -0.56 (-0.63 to -0.49) |
| Eswatini | 368 (285,460) | 101.51 (79.92,124.36) |  | 487 (368,612) | 81.18 (63.01,101.31) | -0.73 (-0.88 to -0.58) |
| Ethiopia | 70632 (54689,86367) | 257.67 (204.94,311.02) |  | 127052 (97379,154664) | 219.53 (172.43,268.5) | -0.76 (-0.94 to -0.59) |
| Fiji | 442 (335,556) | 100.98 (79.49,123.34) |  | 663 (506,836) | 89.82 (69.68,111.28) | -0.44 (-0.57 to -0.31) |
| Finland | 4080 (3188,5220) | 56.73 (44.59,71.26) |  | 6710 (5159,8861) | 52.83 (40.7,67.62) | 0 (-0.09 to 0.08) |
| France | 48223 (36874,60582) | 57.68 (44.69,71.73) |  | 72561 (55758,93556) | 52.68 (40.59,65.36) | -0.35 (-0.49 to -0.2) |
| Gabon | 1066 (824,1304) | 160.85 (127.4,196.48) |  | 1483 (1145,1853) | 126.11 (99.6,156.21) | -0.85 (-0.96 to -0.75) |
| Gambia | 1120 (864,1379) | 206.64 (165.39,252.37) |  | 2253 (1743,2770) | 167.4 (132.43,204.63) | -0.67 (-0.81 to -0.54) |
| Georgia | 6139 (4740,7659) | 102.45 (80.25,125.9) |  | 5412 (4183,6804) | 96.88 (75.16,120.73) | -0.25 (-0.33 to -0.18) |
| Germany | 69079 (52778,87268) | 55.51 (42.87,68.93) |  | 98295 (74422,126151) | 52.02 (40.01,65.84) | -0.26 (-0.37 to -0.15) |
| Ghana | 11675 (8915,14432) | 141.31 (111.04,172.37) |  | 22307 (17097,27827) | 110.77 (85.86,135.28) | -0.8 (-0.89 to -0.7) |
| Greece | 9486 (7303,12095) | 63.81 (49.77,79.46) |  | 13562 (10479,17587) | 57.52 (44.3,71.63) | -0.35 (-0.48 to -0.22) |
| Greenland | 63 (48,78) | 161.48 (125.82,199.57) |  | 93 (70,120) | 144.34 (112.31,180.93) | -0.4 (-0.53 to -0.27) |
| Grenada | 52 (41,65) | 67 (52.09,82.46) |  | 56 (43,70) | 53.47 (41.48,67.35) | -0.75 (-0.84 to -0.66) |
| Guam | 79 (60,100) | 86.85 (68.1,106.82) |  | 157 (121,195) | 77.46 (59.94,96.64) | -0.48 (-0.61 to -0.35) |
| Guatemala | 3293 (2505,4128) | 78.08 (61.58,96.06) |  | 7145 (5503,8772) | 61.82 (48.37,76.74) | -0.81 (-0.9 to -0.73) |
| Guinea | 9089 (7067,11028) | 220.08 (175.55,266.48) |  | 13648 (10649,16884) | 179.94 (141.79,220.69) | -0.62 (-0.76 to -0.49) |
| Guinea-Bissau | 1163 (895,1429) | 213.2 (169.63,258.59) |  | 1863 (1439,2333) | 175.57 (138.58,216.22) | -0.6 (-0.74 to -0.47) |
| Guyana | 294 (225,368) | 68.12 (53.35,83.81) |  | 339 (261,420) | 55.45 (43.11,68.66) | -0.67 (-0.76 to -0.59) |
| Haiti | 2846 (2156,3551) | 79.11 (62.5,97.33) |  | 5126 (3867,6419) | 67.58 (52.95,84.2) | -0.54 (-0.63 to -0.46) |
| Honduras | 1963 (1497,2454) | 76.97 (60.08,94.77) |  | 4192 (3205,5194) | 62.96 (49.07,77.55) | -0.7 (-0.79 to -0.62) |
| Hungary | 13834 (10756,17161) | 101.27 (78.99,125.06) |  | 15777 (12165,19860) | 88.98 (69.33,110.8) | -0.46 (-0.53 to -0.39) |
| Iceland | 176 (136,221) | 58.92 (45.51,73.58) |  | 304 (234,388) | 53.64 (41.26,66.85) | -0.31 (-0.43 to -0.18) |
| India | 831990 (640981,1019138) | 151.03 (119.66,184.28) |  | 1378441 (1063758,1709069) | 113.04 (89.11,138.46) | -0.97 (-1.13 to -0.82) |
| Indonesia | 180451 (137083,225488) | 145.94 (114.07,179.15) |  | 292192 (220829,367430) | 120.18 (93.26,147.11) | -0.74 (-0.86 to -0.62) |
| Iran (Islamic Republic of) | 107601 (82177,134755) | 277.83 (219.12,336.18) |  | 156826 (121012,192757) | 192.56 (151.03,232.61) | -1.31 (-1.36 to -1.26) |
| Iraq | 33629 (26456,41459) | 279.23 (219.93,340.98) |  | 57151 (44126,71341) | 188.44 (147.82,228.36) | -1.43 (-1.48 to -1.39) |
| Ireland | 2491 (1915,3159) | 61.18 (47.5,76.64) |  | 4069 (3076,5153) | 53.51 (41.25,65.82) | -0.46 (-0.59 to -0.33) |
| Israel | 3179 (2447,4020) | 62.06 (48.27,77.7) |  | 6990 (5376,8859) | 57.26 (44.17,71.7) | -0.26 (-0.39 to -0.14) |
| Italy | 65717 (50750,82420) | 75.64 (59.1,94.07) |  | 95676 (73525,123857) | 69.04 (53.73,86.39) | -0.28 (-0.37 to -0.2) |
| Jamaica | 1245 (975,1524) | 62.29 (48.58,76.54) |  | 1672 (1299,2081) | 52.61 (40.65,66) | -0.58 (-0.67 to -0.49) |
| Japan | 163123 (125827,204446) | 96.22 (74.5,119.58) |  | 306504 (234521,397996) | 88.55 (68.48,110.39) | -0.24 (-0.39 to -0.09) |
| Jordan | 5711 (4412,7215) | 244.84 (193.9,295.57) |  | 16810 (12719,20792) | 179.17 (140.09,220.31) | -1.15 (-1.2 to -1.1) |
| Kazakhstan | 13790 (10613,17061) | 106.79 (82.9,131.4) |  | 16250 (12451,20495) | 95.44 (73.96,118.35) | -0.42 (-0.49 to -0.36) |
| Kenya | 32526 (25449,39464) | 258.36 (204.87,313.26) |  | 67959 (52024,82426) | 229.79 (181.26,281.42) | -0.57 (-0.72 to -0.43) |
| Kiribati | 49 (37,62) | 113.71 (89.83,138.69) |  | 80 (60,101) | 103.58 (81.32,128.01) | -0.35 (-0.48 to -0.22) |
| Kuwait | 2356 (1775,2946) | 215.87 (169.88,261.94) |  | 6022 (4470,7531) | 157.23 (122.7,191.8) | -1.14 (-1.19 to -1.1) |
| Kyrgyzstan | 3687 (2848,4526) | 113.25 (89.14,139.07) |  | 5296 (4028,6537) | 106.39 (82.95,131.23) | -0.23 (-0.3 to -0.16) |
| Lao People's Democratic Republic | 4885 (3744,6104) | 181.01 (142.23,221.66) |  | 7860 (5959,9830) | 145.26 (113.45,178.62) | -0.84 (-0.96 to -0.71) |
| Latvia | 3357 (2594,4178) | 99.27 (76.96,122.17) |  | 3194 (2449,4044) | 87.06 (67.59,108.05) | -0.49 (-0.56 to -0.41) |
| Lebanon | 6526 (5074,8026) | 259.37 (204.64,314.16) |  | 10924 (8603,13222) | 181.37 (141.32,219.44) | -1.3 (-1.35 to -1.26) |
| Lesotho | 1028 (796,1271) | 110.06 (86.82,137) |  | 966 (738,1209) | 87.15 (68.48,108.67) | -0.76 (-0.91 to -0.61) |
| Liberia | 2815 (2205,3429) | 214 (169.85,259.19) |  | 5270 (4009,6585) | 173.92 (138,213.37) | -0.73 (-0.86 to -0.59) |
| Libya | 7137 (5568,8920) | 254.39 (199.47,310.43) |  | 10838 (8236,13297) | 181.72 (141.83,220.28) | -1.23 (-1.31 to -1.15) |
| Lithuania | 4438 (3447,5476) | 100.9 (78.4,124.35) |  | 4480 (3468,5688) | 83.9 (64.87,103.24) | -0.67 (-0.74 to -0.6) |
| Luxembourg | 308 (237,392) | 57.37 (44.64,71.85) |  | 545 (418,694) | 52.65 (40.47,66.12) | -0.3 (-0.43 to -0.18) |
| Madagascar | 19130 (15202,23523) | 265.96 (216.28,319.98) |  | 39663 (30756,49489) | 250.03 (199.02,303.42) | -0.52 (-0.65 to -0.38) |
| Malawi | 11439 (8773,13853) | 227.53 (178.89,278.25) |  | 17598 (13836,21519) | 184.93 (147.29,226.15) | -0.79 (-1.02 to -0.56) |
| Malaysia | 16923 (13094,21167) | 135.9 (105.84,168.49) |  | 33409 (25615,41996) | 114.14 (88.73,142.77) | -0.64 (-0.76 to -0.51) |
| Maldives | 217 (165,272) | 166.37 (130.86,204.52) |  | 531 (398,664) | 126.1 (98.17,156.67) | -1 (-1.13 to -0.88) |
| Mali | 11798 (9153,14534) | 232.16 (186.42,282.77) |  | 23783 (18170,29498) | 189.62 (148.41,230.51) | -0.65 (-0.78 to -0.52) |
| Malta | 277 (212,350) | 64.85 (50.43,81.25) |  | 530 (405,689) | 57.21 (44.13,72.06) | -0.43 (-0.56 to -0.3) |
| Marshall Islands | 25 (19,31) | 111.22 (87.18,135.62) |  | 37 (27,46) | 99.64 (77.19,123.04) | -0.38 (-0.51 to -0.25) |
| Mauritania | 2427 (1870,2978) | 190.47 (150.75,234.84) |  | 4089 (3178,5075) | 152.46 (121.04,189.21) | -0.71 (-0.84 to -0.57) |
| Mauritius | 1181 (911,1471) | 136.38 (106.91,169.6) |  | 1935 (1495,2435) | 116.56 (91.54,143.41) | -0.59 (-0.71 to -0.46) |
| Mexico | 35941 (27422,45250) | 70.11 (54.61,86.61) |  | 73242 (56197,90135) | 59.32 (45.86,73.49) | -0.58 (-0.67 to -0.5) |
| Micronesia (Federated States of) | 63 (49,80) | 107.99 (84.21,132.52) |  | 72 (54,91) | 97.48 (75.64,119.27) | -0.39 (-0.52 to -0.26) |
| Monaco | 38 (29,50) | 54.49 (42.05,68.62) |  | 48 (37,62) | 51.41 (39.79,64.59) | -0.2 (-0.33 to -0.07) |
| Mongolia | 1556 (1188,1922) | 122.85 (96.18,150.02) |  | 2526 (1917,3151) | 107.17 (82.74,132.33) | -0.49 (-0.55 to -0.42) |
| Montenegro | 635 (488,789) | 100.35 (77.45,123.6) |  | 785 (601,995) | 89.56 (69.9,111.15) | -0.49 (-0.56 to -0.42) |
| Morocco | 54522 (42366,67516) | 286.82 (226.61,348.98) |  | 72609 (55646,89246) | 207.72 (161.77,255.26) | -1.14 (-1.18 to -1.09) |
| Mozambique | 17694 (13907,21476) | 235.63 (188.58,283.15) |  | 30893 (24037,37295) | 209.19 (164.9,257.09) | -0.64 (-0.82 to -0.47) |
| Myanmar | 58205 (44267,72231) | 197.3 (155.03,240.24) |  | 79738 (61249,100394) | 157.3 (122.37,195.76) | -0.95 (-1.09 to -0.81) |
| Namibia | 723 (551,904) | 96.8 (75.86,119.83) |  | 1135 (853,1418) | 78.85 (60.53,98.23) | -0.7 (-0.85 to -0.55) |
| Nauru | 6 (4,7) | 102.09 (79.54,124.9) |  | 6 (5,8) | 92.53 (72.42,114.2) | -0.42 (-0.55 to -0.28) |
| Nepal | 21649 (17646,25857) | 183.61 (148.89,222.04) |  | 30860 (23728,37854) | 126.62 (99.63,155.08) | -1.27 (-1.42 to -1.13) |
| Netherlands | 11499 (8847,14467) | 57.05 (44.53,71.43) |  | 17537 (13272,22715) | 52.56 (40.38,66.25) | -0.28 (-0.41 to -0.16) |
| New Zealand | 4897 (3779,6094) | 122.42 (95.02,152.53) |  | 8848 (6850,11090) | 111.47 (86.67,139.21) | -0.3 (-0.41 to -0.19) |
| Nicaragua | 1546 (1201,1922) | 76.18 (59.54,93.61) |  | 3144 (2419,3886) | 62.09 (48.18,77.12) | -0.68 (-0.77 to -0.59) |
| Niger | 10447 (8062,12975) | 245.03 (195.57,295.03) |  | 27263 (21506,33899) | 218.5 (174.02,267.24) | -0.33 (-0.47 to -0.2) |
| Nigeria | 123466 (94995,152188) | 212.14 (168.55,258.84) |  | 207949 (160383,257246) | 162.56 (127.97,199.48) | -0.99 (-1.17 to -0.8) |
| Niue | 2 (2,3) | 97.29 (76.59,119.92) |  | 2 (1,2) | 85.36 (65.7,106.19) | -0.52 (-0.65 to -0.39) |
| North Macedonia | 1978 (1522,2454) | 106.61 (83.26,130.89) |  | 2710 (2050,3453) | 92.56 (71.81,114.77) | -0.54 (-0.6 to -0.47) |
| Northern Mariana Islands | 21 (15,27) | 82.5 (64.51,101.75) |  | 37 (27,47) | 78.59 (61.02,96.59) | -0.14 (-0.27 to -0.01) |
| Norway | 4476 (3432,5721) | 66.57 (51.34,83.26) |  | 5819 (4528,7357) | 60.81 (47.18,76.08) | -0.28 (-0.47 to -0.09) |
| Oman | 3239 (2501,4102) | 267.06 (214.64,321.33) |  | 5682 (4247,7245) | 174.37 (137.13,213.14) | -1.51 (-1.57 to -1.45) |
| Pakistan | 91796 (72601,112510) | 138.13 (109.4,169.3) |  | 149464 (114143,182734) | 108.01 (84.88,132.56) | -0.88 (-1.02 to -0.74) |
| Palau | 10 (8,13) | 90.04 (69.87,110.82) |  | 16 (12,21) | 81.77 (63.37,101.11) | -0.35 (-0.48 to -0.22) |
| Palestine | 4010 (3139,5032) | 298.71 (235.89,363.77) |  | 7323 (5648,9124) | 206.53 (161.94,251.8) | -1.31 (-1.36 to -1.27) |
| Panama | 1071 (821,1319) | 62.05 (48,76.13) |  | 2321 (1811,2896) | 52.12 (40.32,65.14) | -0.59 (-0.68 to -0.5) |
| Papua New Guinea | 2893 (2182,3631) | 125.59 (98.35,153.51) |  | 6790 (5130,8587) | 113.64 (89.2,139.83) | -0.36 (-0.49 to -0.23) |
| Paraguay | 1236 (957,1556) | 47.48 (37.32,59.82) |  | 2206 (1725,2794) | 37.52 (29.56,47.49) | -0.8 (-0.97 to -0.63) |
| Peru | 7896 (6088,9704) | 57.12 (44.61,70.59) |  | 16221 (12571,20354) | 47.46 (36.51,59.95) | -0.65 (-0.76 to -0.54) |
| Philippines | 63534 (48397,78815) | 154.57 (121.32,190.34) |  | 124955 (95659,156541) | 138.44 (108.65,171.28) | -0.4 (-0.52 to -0.28) |
| Poland | 47918 (36896,59163) | 112.87 (87.52,137.72) |  | 62051 (47620,76904) | 95.51 (75.2,116.82) | -0.59 (-0.66 to -0.52) |
| Portugal | 9224 (7049,11664) | 68.38 (53.31,84.73) |  | 14313 (10973,18521) | 60.29 (46.93,75.16) | -0.43 (-0.56 to -0.3) |
| Puerto Rico | 2079 (1590,2615) | 54.84 (42.45,68.45) |  | 3248 (2455,4161) | 45.5 (35.07,57.7) | -0.65 (-0.74 to -0.57) |
| Qatar | 632 (470,825) | 223.09 (174.91,273.31) |  | 3256 (2374,4317) | 157.83 (122.43,192.8) | -1.17 (-1.23 to -1.12) |
| Republic of Korea | 30671 (23192,38439) | 95.77 (74.89,118.18) |  | 69411 (53071,88498) | 79.83 (61.71,99.85) | -0.55 (-0.7 to -0.4) |
| Republic of Moldova | 4669 (3568,5803) | 110.26 (85.91,134.99) |  | 5289 (4070,6638) | 96.53 (74.93,119.69) | -0.51 (-0.58 to -0.43) |
| Romania | 27492 (21022,34065) | 104.61 (81.16,127.82) |  | 31331 (24186,39741) | 92.46 (71.2,114.22) | -0.49 (-0.56 to -0.42) |
| Russian Federation | 186420 (142293,231507) | 108.2 (84.1,131.89) |  | 209334 (160602,259093) | 95.85 (75.31,117.13) | -0.45 (-0.53 to -0.38) |
| Rwanda | 7820 (6095,9545) | 208.38 (165.7,252.66) |  | 14192 (10817,17426) | 186.76 (147.13,228.95) | -0.66 (-0.84 to -0.48) |
| Saint Kitts and Nevis | 23 (17,28) | 59.32 (46.05,74.25) |  | 30 (23,38) | 49.11 (38.7,62.26) | -0.65 (-0.74 to -0.56) |
| Saint Lucia | 63 (49,78) | 65.48 (51.02,80.72) |  | 121 (92,154) | 53.32 (41.03,67.37) | -0.68 (-0.77 to -0.59) |
| Saint Vincent and the Grenadines | 53 (41,66) | 67.44 (52.69,82.69) |  | 74 (56,93) | 55.49 (42.49,68.55) | -0.64 (-0.73 to -0.55) |
| Samoa | 99 (77,125) | 100.12 (78.9,121.98) |  | 137 (106,172) | 91.33 (71.19,112.82) | -0.34 (-0.47 to -0.21) |
| San Marino | 21 (16,26) | 56.18 (43.46,70.21) |  | 39 (30,51) | 52.99 (40.83,66.58) | -0.21 (-0.34 to -0.08) |
| Sao Tome and Principe | 152 (120,186) | 196.63 (156.91,240.77) |  | 211 (161,263) | 153.01 (121.51,186.71) | -0.85 (-0.99 to -0.71) |
| Saudi Arabia | 25145 (19363,31638) | 246.09 (194.94,299.6) |  | 45223 (33562,57442) | 162.12 (126.94,197.67) | -1.51 (-1.56 to -1.46) |
| Senegal | 9167 (7145,11244) | 206.83 (164.87,252.63) |  | 16235 (12490,19849) | 166.34 (131.32,204.99) | -0.67 (-0.81 to -0.54) |
| Serbia | 10836 (8240,13594) | 104 (81.13,128.12) |  | 13453 (10198,16804) | 89.8 (69.45,110.68) | -0.58 (-0.65 to -0.51) |
| Seychelles | 83 (64,103) | 130.93 (101.95,161.72) |  | 128 (99,161) | 114.47 (89.51,142.43) | -0.46 (-0.59 to -0.34) |
| Sierra Leone | 5638 (4416,6870) | 219.64 (172.49,269.39) |  | 9313 (7055,11598) | 181.89 (140.61,223.65) | -0.62 (-0.79 to -0.45) |
| Singapore | 2381 (1821,2969) | 96.19 (74.63,119.23) |  | 6565 (5069,8248) | 81.1 (63.15,101.06) | -0.58 (-0.73 to -0.43) |
| Slovakia | 5889 (4520,7321) | 100.6 (77.52,124.17) |  | 7620 (5860,9636) | 88.45 (68.77,109.89) | -0.48 (-0.55 to -0.4) |
| Slovenia | 2296 (1786,2846) | 94.83 (73.72,116.84) |  | 3410 (2596,4351) | 84.22 (65.55,104.52) | -0.4 (-0.47 to -0.33) |
| Solomon Islands | 230 (173,289) | 127.22 (100.16,154.84) |  | 459 (346,571) | 114.76 (88.79,140.85) | -0.38 (-0.51 to -0.26) |
| Somalia | 9242 (7244,11461) | 255.47 (203.32,307.94) |  | 24609 (19129,30634) | 263.58 (206.89,321.65) | -0.09 (-0.27 to 0.08) |
| South Africa | 27074 (21019,33388) | 112.5 (88.47,138) |  | 43718 (33684,54308) | 94.73 (74.54,117.12) | -0.61 (-0.69 to -0.52) |
| South Sudan | 7257 (5703,8825) | 220.03 (174.88,266.32) |  | 10670 (8317,13169) | 216.3 (170.8,264.52) | -0.24 (-0.42 to -0.07) |
| Spain | 35977 (27583,45682) | 66.1 (51.23,82.21) |  | 56560 (43169,72596) | 59.16 (45.74,73.92) | -0.37 (-0.5 to -0.24) |
| Sri Lanka | 18584 (14367,23195) | 141.56 (110.9,174.88) |  | 30349 (23301,37699) | 120.31 (94.35,148.9) | -0.69 (-0.81 to -0.56) |
| Sudan | 45282 (35630,56354) | 325.43 (257.78,396.73) |  | 62204 (47807,78152) | 214.44 (168.08,262.27) | -1.5 (-1.54 to -1.45) |
| Suriname | 178 (138,222) | 64.64 (50.21,79.68) |  | 333 (256,417) | 55.25 (42.65,69.52) | -0.59 (-0.68 to -0.5) |
| Sweden | 8705 (6681,11044) | 60.84 (47.4,76.04) |  | 10835 (8355,13993) | 54.81 (42.61,68.92) | -0.42 (-0.63 to -0.21) |
| Switzerland | 5644 (4342,7187) | 53.39 (41.47,66.35) |  | 8984 (6945,11536) | 50.5 (39.16,63.37) | -0.21 (-0.34 to -0.08) |
| Syrian Arab Republic | 23035 (18148,28803) | 281.19 (223.12,341.88) |  | 25273 (19537,31726) | 192.84 (151.7,236.75) | -1.34 (-1.4 to -1.28) |
| Taiwan (Province of China) | 9168 (7047,11388) | 55.87 (44.06,69.34) |  | 18843 (14535,24066) | 47.49 (36.72,59.49) | -0.64 (-0.91 to -0.38) |
| Tajikistan | 3960 (3069,4893) | 121.74 (94.69,148.88) |  | 7206 (5316,8973) | 112.78 (87.26,137.42) | -0.33 (-0.41 to -0.26) |
| Thailand | 63100 (48923,78681) | 141.99 (112.52,176.03) |  | 117373 (90786,147365) | 121.35 (94.76,150.78) | -0.57 (-0.69 to -0.44) |
| Timor-Leste | 796 (604,1014) | 177.66 (140.24,217.42) |  | 1401 (1076,1755) | 141.92 (111.54,175.42) | -0.91 (-1.03 to -0.78) |
| Togo | 3857 (2972,4740) | 201.97 (160.1,246.88) |  | 8234 (6295,10265) | 167.15 (132.39,206.69) | -0.57 (-0.71 to -0.43) |
| Tokelau | 1 (1,2) | 109.35 (85.78,134.29) |  | 1 (1,2) | 91.46 (71.27,112.97) | -0.68 (-0.81 to -0.55) |
| Tonga | 64 (49,80) | 101.88 (80,124.47) |  | 77 (59,94) | 91.52 (70.59,112.62) | -0.39 (-0.52 to -0.26) |
| Trinidad and Tobago | 527 (407,648) | 57.63 (44.65,71.38) |  | 870 (667,1106) | 48.02 (37.14,60.53) | -0.67 (-0.75 to -0.58) |
| Tunisia | 17117 (13257,21132) | 263.23 (207.4,316.68) |  | 23243 (17920,28535) | 185.01 (143.31,226.08) | -1.23 (-1.28 to -1.18) |
| Turkey | 117842 (91673,146350) | 263.78 (208.02,319) |  | 160583 (124792,197731) | 179.83 (140.67,219.59) | -1.37 (-1.41 to -1.32) |
| Turkmenistan | 2523 (1928,3126) | 112.24 (87.67,138.33) |  | 4024 (3079,5010) | 99.95 (77.09,123.18) | -0.46 (-0.53 to -0.4) |
| Tuvalu | 8 (6,9) | 111.48 (87.44,136.71) |  | 10 (8,12) | 96.49 (74.82,118.66) | -0.5 (-0.63 to -0.37) |
| Uganda | 14267 (11114,17433) | 164.86 (130.13,200.65) |  | 27508 (21179,34092) | 144.37 (113.16,178.22) | -0.77 (-1.07 to -0.48) |
| Ukraine | 73321 (56316,91895) | 110.89 (86.74,136.4) |  | 71865 (55356,90070) | 101.82 (80.14,125.04) | -0.36 (-0.43 to -0.28) |
| United Arab Emirates | 2649 (1996,3468) | 218.06 (171.51,264.42) |  | 11431 (8152,15084) | 154.05 (119.83,188.78) | -1.19 (-1.25 to -1.13) |
| United Kingdom | 81095 (62798,101993) | 93.16 (72.76,115.61) |  | 99378 (76490,123874) | 81.67 (63.48,101.2) | 0.08 (-0.02 to 0.18) |
| United Republic of Tanzania | 22884 (17595,28092) | 167.58 (131.93,206.9) |  | 46856 (36450,57795) | 154.91 (119.35,192.1) | -0.44 (-0.62 to -0.26) |
| United States of America | 552188 (433369,685990) | 173.56 (134.7,214.96) |  | 832578 (645771,1050193) | 157.49 (123.46,195.65) | -0.32 (-0.38 to -0.26) |
| United States Virgin Islands | 46 (35,58) | 53.76 (41.83,66.73) |  | 77 (58,102) | 45.91 (35.26,57.99) | -0.54 (-0.63 to -0.45) |
| Uruguay | 4439 (3472,5528) | 115.64 (90.59,142.54) |  | 5502 (4264,6894) | 103.51 (81.17,127.81) | -0.38 (-0.42 to -0.35) |
| Uzbekistan | 16379 (12676,20127) | 118.89 (92.77,146.42) |  | 27038 (20644,33471) | 102.99 (80.16,127.13) | -0.53 (-0.59 to -0.46) |
| Vanuatu | 97 (72,120) | 118.22 (93.1,144.45) |  | 205 (155,258) | 107.12 (83.63,131.59) | -0.39 (-0.52 to -0.27) |
| Venezuela (Bolivarian Republic of) | 7427 (5715,9289) | 63.61 (49.59,79.07) |  | 16182 (12470,20062) | 56.67 (43.94,69.98) | -0.42 (-0.51 to -0.33) |
| Viet Nam | 79063 (61450,98729) | 158.87 (124.15,195.73) |  | 126177 (97203,159010) | 128.23 (100.38,159.91) | -0.79 (-0.91 to -0.67) |
| Yemen | 28148 (21975,35287) | 339.47 (267.3,412.93) |  | 50594 (39219,63837) | 232.63 (184.16,283.34) | -1.38 (-1.43 to -1.32) |
| Zambia | 7998 (6187,9698) | 201.5 (158.46,243.19) |  | 16466 (12838,20564) | 176.4 (139.98,215.69) | -0.72 (-0.9 to -0.54) |
| Zimbabwe | 4493 (3475,5549) | 90.14 (70.82,111.2) |  | 6088 (4616,7699) | 80.21 (62.2,99.44) | -0.27 (-0.47 to -0.07) |
| ASPR, age-standardized prevalence rate. EAPC, estimated annual percentage changes. UI, uncertainty interval. CI, confidence interval. | | | | | | |

| **Table S2** The prevalence rate of complete hearing loss in 1992 and 2021, and its relative change from 1992-2021 among 204 countries and territories. | | | |
| --- | --- | --- | --- |
| **Characteristics** | **Prevalence rate  in 1992** | **Prevalence rate  in 2021** | **1992-2021** |
|  |  |  | **Relative change (%)** |
| Afghanistan | 255.77 (200.44,313.39) | 150.58 (115.88,191.44) | -41.13 |
| Albania | 80.7 (61.24,100.85) | 145.98 (112.32,183.9) | 80.89 |
| Algeria | 171.96 (132.65,214.47) | 163.65 (127.02,200.83) | -4.83 |
| American Samoa | 49.78 (37.23,62.86) | 76.29 (57.13,96.85) | 53.25 |
| Andorra | 57.13 (43.8,71.69) | 90.95 (70.24,115.37) | 59.20 |
| Angola | 94.27 (72.85,116.19) | 71.22 (54.14,88.26) | -24.45 |
| Antigua and Barbuda | 55.4 (43.2,68.37) | 54.45 (41.88,68.95) | -1.71 |
| Argentina | 110.02 (85.25,136.11) | 121.89 (94.38,152.15) | 10.79 |
| Armenia | 94.85 (72.61,118.68) | 133.29 (102.63,168.14) | 40.53 |
| Australia | 136.2 (107.47,166.61) | 177.4 (135.36,225.39) | 30.25 |
| Austria | 88.23 (67.34,113.05) | 107.6 (82.57,139.23) | 21.95 |
| Azerbaijan | 80.09 (61.37,99.76) | 91.41 (68.65,114.18) | 14.13 |
| Bahamas | 35.58 (27.45,44.32) | 45.75 (34.91,57.84) | 28.58 |
| Bahrain | 146.09 (110.18,186) | 136.24 (101.38,174.3) | -6.74 |
| Bangladesh | 66.78 (51.96,82.11) | 84.67 (65.39,103.87) | 26.79 |
| Barbados | 66.91 (51.2,85.59) | 79.76 (60.74,102.34) | 19.20 |
| Belarus | 124.14 (95.8,154.88) | 142.82 (109.84,180.04) | 15.05 |
| Belgium | 90.23 (68.61,115.1) | 108.07 (82.92,139.59) | 19.77 |
| Belize | 40.09 (31.17,49.17) | 40.45 (31.17,49.68) | 0.90 |
| Benin | 126.47 (99.74,154.83) | 104.14 (81.11,128.58) | -17.66 |
| Bermuda | 56 (43.38,70.64) | 92.9 (69.8,118.4) | 65.89 |
| Bhutan | 74.05 (56.94,92.44) | 92.86 (72.49,114.09) | 25.40 |
| Bolivia (Plurinational State of) | 45.67 (34.68,56.49) | 52.24 (39.7,65) | 14.39 |
| Bosnia and Herzegovina | 110.1 (85.09,137.3) | 160.29 (124.33,202.16) | 45.59 |
| Botswana | 46.94 (35.79,58.7) | 48.47 (36.44,61.09) | 3.26 |
| Brazil | 33.14 (25.44,41.26) | 44.97 (35.22,56.46) | 35.70 |
| Brunei Darussalam | 47.93 (36.32,59.6) | 64.81 (48.45,80.89) | 35.22 |
| Bulgaria | 134.28 (101.3,169.84) | 172.42 (130.17,219.23) | 28.40 |
| Burkina Faso | 131.09 (102.05,159.92) | 105.72 (80.95,130.16) | -19.35 |
| Burundi | 113.98 (89.45,138.51) | 106.52 (82.27,130.16) | -6.55 |
| Cabo Verde | 144.08 (115.8,176.7) | 128.77 (99.76,158.44) | -10.63 |
| Cambodia | 105.34 (81.7,131.39) | 118.65 (90.14,148.23) | 12.64 |
| Cameroon | 120.15 (92.83,147.13) | 96.25 (73.99,119.75) | -19.89 |
| Canada | 175.26 (136.36,219.48) | 232 (177.96,294.71) | 32.37 |
| Central African Republic | 102.84 (78.66,126.26) | 92.39 (69.99,115.05) | -10.16 |
| Chad | 135.36 (105.85,165.4) | 97.51 (75.53,120.53) | -27.96 |
| Chile | 91.47 (70.75,113.59) | 127.3 (98.62,158.95) | 39.17 |
| China | 101.45 (77.11,126.77) | 164.85 (126.33,209.01) | 62.49 |
| Colombia | 40.68 (31.49,50.79) | 62.05 (48.34,77.71) | 52.53 |
| Comoros | 107.01 (83.1,130.08) | 128.07 (99.84,157.92) | 19.68 |
| Congo | 89.27 (69.15,110) | 80.47 (61.11,99.8) | -9.86 |
| Cook Islands | 64.38 (49.08,80.62) | 107.42 (82.06,134.78) | 66.85 |
| Costa Rica | 40.75 (31.42,50.49) | 59.99 (46.33,75.31) | 47.21 |
| Croatia | 119.58 (91.69,149.81) | 169.63 (128.73,213.24) | 41.85 |
| Cuba | 59.14 (45.12,73.34) | 87.45 (67.21,111.5) | 47.87 |
| Cyprus | 64.11 (48.12,81.72) | 79.95 (61.3,102.42) | 24.71 |
| Czechia | 122.54 (93.22,153.7) | 158.24 (121.21,202.7) | 29.13 |
| C么te d'Ivoire | 98.6 (75.51,121.64) | 91.15 (69.66,113.73) | -7.56 |
| Democratic People's Republic of Korea | 73.45 (56.25,91.87) | 100.28 (77.29,124.94) | 36.53 |
| Democratic Republic of the Congo | 98.09 (75.89,119.57) | 86.16 (65.3,107) | -12.16 |
| Denmark | 88.04 (67.15,112.27) | 101.87 (77.48,130.91) | 15.71 |
| Djibouti | 96.41 (74.01,118.46) | 112.8 (86.65,139.91) | 17.00 |
| Dominica | 52.37 (40.43,65.02) | 58.22 (45.16,73.92) | 11.17 |
| Dominican Republic | 41.92 (32.17,51.91) | 52.19 (40.87,64.79) | 24.50 |
| Ecuador | 60.69 (47.15,74.13) | 75.43 (57.6,92.09) | 24.29 |
| Egypt | 179.6 (138.81,224.22) | 138.6 (106.2,175.58) | -22.83 |
| El Salvador | 46.98 (36.26,57.77) | 59.06 (45.61,73.15) | 25.71 |
| Equatorial Guinea | 103.22 (79.29,126.56) | 58.94 (44.49,74.63) | -42.90 |
| Eritrea | 99.58 (77.12,124.24) | 100.64 (77.67,124.77) | 1.06 |
| Estonia | 125.53 (97.29,155.52) | 163.56 (124.63,208.29) | 30.30 |
| Eswatini | 43 (33.27,53.77) | 42.18 (31.89,52.94) | -1.91 |
| Ethiopia | 128.7 (99.65,157.37) | 116.63 (89.39,141.97) | -9.38 |
| Fiji | 57.39 (43.48,72.15) | 71.73 (54.7,90.41) | 24.99 |
| Finland | 80.68 (63.03,103.22) | 121.22 (93.18,160.07) | 50.25 |
| France | 82.92 (63.4,104.17) | 109.3 (83.98,140.92) | 31.81 |
| Gabon | 103.4 (79.89,126.48) | 81.65 (63.02,102.01) | -21.03 |
| Gambia | 105.45 (81.32,129.83) | 94.09 (72.79,115.72) | -10.77 |
| Georgia | 112.82 (87.11,140.76) | 150.03 (115.96,188.62) | 32.98 |
| Germany | 85.46 (65.3,107.97) | 115.14 (87.17,147.77) | 34.73 |
| Ghana | 74.36 (56.78,91.92) | 65.14 (49.92,81.26) | -12.40 |
| Greece | 89.81 (69.15,114.52) | 133.28 (102.99,172.85) | 48.40 |
| Greenland | 113.18 (86.15,141.29) | 166.34 (124.37,213.38) | 46.97 |
| Grenada | 59.35 (46.46,73.4) | 54.22 (41.76,68.66) | -8.64 |
| Guam | 55.61 (41.78,70.2) | 98.35 (75.94,122.78) | 76.86 |
| Guatemala | 37.1 (28.22,46.51) | 45.31 (34.9,55.63) | 22.13 |
| Guinea | 139.92 (108.8,169.77) | 101.62 (79.29,125.72) | -27.37 |
| Guinea-Bissau | 110.59 (85.09,135.91) | 90.26 (69.71,113.04) | -18.38 |
| Guyana | 37.77 (28.91,47.22) | 44.34 (34.08,54.96) | 17.39 |
| Haiti | 42.42 (32.13,52.93) | 39.85 (30.06,49.9) | -6.06 |
| Honduras | 39.37 (30.01,49.22) | 41.46 (31.7,51.37) | 5.31 |
| Hungary | 133.43 (103.74,165.52) | 164.39 (126.75,206.94) | 23.20 |
| Iceland | 67.74 (52.59,85.3) | 86.76 (66.76,110.64) | 28.08 |
| India | 93.98 (72.4,115.12) | 97.45 (75.2,120.83) | 3.69 |
| Indonesia | 94.74 (71.97,118.39) | 104.76 (79.17,131.74) | 10.58 |
| Iran (Islamic Republic of) | 178.91 (136.64,224.06) | 183.73 (141.77,225.83) | 2.69 |
| Iraq | 176.44 (138.81,217.52) | 138.63 (107.04,173.06) | -21.43 |
| Ireland | 68.97 (53.03,87.47) | 82.35 (62.26,104.29) | 19.40 |
| Israel | 60.66 (46.68,76.7) | 72.85 (56.04,92.34) | 20.10 |
| Italy | 115.89 (89.49,145.34) | 159.96 (122.93,207.08) | 38.03 |
| Jamaica | 51.72 (40.5,63.28) | 59.7 (46.4,74.32) | 15.43 |
| Japan | 128.86 (99.4,161.5) | 240.02 (183.65,311.67) | 86.26 |
| Jordan | 141.92 (109.66,179.3) | 136.39 (103.2,168.7) | -3.90 |
| Kazakhstan | 85.08 (65.48,105.26) | 85.73 (65.69,108.12) | 0.76 |
| Kenya | 132.08 (103.34,160.26) | 135.75 (103.92,164.65) | 2.78 |
| Kiribati | 64.1 (48.64,80.56) | 65.77 (49.85,83.25) | 2.61 |
| Kuwait | 138.93 (104.67,173.74) | 129.5 (96.13,161.95) | -6.79 |
| Kyrgyzstan | 80.14 (61.9,98.37) | 77.17 (58.68,95.25) | -3.71 |
| Lao People's Democratic Republic | 111.24 (85.26,138.99) | 106.54 (80.77,133.25) | -4.23 |
| Latvia | 127.44 (98.49,158.61) | 170.8 (130.96,216.2) | 34.02 |
| Lebanon | 210.6 (163.74,259) | 197.16 (155.28,238.64) | -6.38 |
| Lesotho | 65.2 (50.46,80.61) | 51.56 (39.35,64.48) | -20.92 |
| Liberia | 140.98 (110.45,171.72) | 96.54 (73.43,120.62) | -31.52 |
| Libya | 164.85 (128.61,206.02) | 157.74 (119.87,193.54) | -4.31 |
| Lithuania | 120.32 (93.44,148.46) | 164.2 (127.1,208.47) | 36.47 |
| Luxembourg | 79.29 (60.85,100.93) | 84.53 (64.89,107.76) | 6.61 |
| Madagascar | 152.29 (121.02,187.26) | 138.89 (107.7,173.3) | -8.80 |
| Malawi | 109.1 (83.67,132.12) | 90.49 (71.14,110.65) | -17.06 |
| Malaysia | 90.16 (69.76,112.77) | 105.02 (80.52,132.01) | 16.48 |
| Maldives | 91.93 (69.98,115.06) | 102.74 (76.98,128.44) | 11.76 |
| Mali | 131.04 (101.66,161.43) | 98.67 (75.38,122.38) | -24.70 |
| Malta | 73.39 (56.12,92.61) | 119.87 (91.51,155.84) | 63.33 |
| Marshall Islands | 52.21 (39.87,65.76) | 65.02 (48.72,82.47) | 24.54 |
| Mauritania | 112.3 (86.51,137.77) | 93.01 (72.29,115.45) | -17.18 |
| Mauritius | 105.33 (81.19,131.15) | 152.13 (117.51,191.42) | 44.43 |
| Mexico | 40.45 (30.87,50.93) | 56.66 (43.47,69.72) | 40.07 |
| Micronesia (Federated States of) | 59.73 (45.83,75.32) | 70.02 (52.25,88.44) | 17.23 |
| Monaco | 122.7 (93.39,161) | 127.13 (96.88,164.82) | 3.61 |
| Mongolia | 69.59 (53.1,85.93) | 75.73 (57.46,94.46) | 8.82 |
| Montenegro | 100.81 (77.45,125.27) | 127.03 (97.3,161.02) | 26.01 |
| Morocco | 207.44 (161.19,256.87) | 195.31 (149.68,240.06) | -5.85 |
| Mozambique | 130.43 (102.51,158.31) | 99.42 (77.36,120.02) | -23.78 |
| Myanmar | 140.38 (106.76,174.21) | 141.33 (108.56,177.94) | 0.68 |
| Namibia | 48.38 (36.87,60.46) | 46.68 (35.08,58.33) | -3.51 |
| Nauru | 54.37 (40.6,68.38) | 54.18 (40.97,69.43) | -0.35 |
| Nepal | 106.68 (86.96,127.42) | 99.13 (76.22,121.6) | -7.08 |
| Netherlands | 76.01 (58.48,95.63) | 101.89 (77.12,131.98) | 34.05 |
| New Zealand | 140.3 (108.26,174.58) | 171.16 (132.51,214.53) | 22.00 |
| Nicaragua | 37.48 (29.11,46.6) | 47.14 (36.28,58.28) | 25.77 |
| Niger | 122.21 (94.32,151.78) | 108.88 (85.89,135.39) | -10.91 |
| Nigeria | 129.25 (99.45,159.32) | 89.96 (69.38,111.28) | -30.40 |
| Niue | 96.69 (76.42,118.66) | 102.05 (77.97,127.69) | 5.54 |
| North Macedonia | 99.2 (76.31,123.02) | 124.53 (94.21,158.63) | 25.53 |
| Northern Mariana Islands | 43.28 (31.86,55.75) | 75.44 (56.25,96.35) | 74.31 |
| Norway | 104.44 (80.07,133.49) | 107.39 (83.57,135.79) | 2.82 |
| Oman | 155.36 (119.98,196.75) | 120.8 (90.29,154.03) | -22.25 |
| Pakistan | 79.11 (62.57,96.96) | 63.45 (48.46,77.58) | -19.80 |
| Palau | 63.46 (47.95,79.16) | 88.85 (65.58,113.93) | 40.01 |
| Palestine | 181.41 (142.01,227.62) | 142.59 (109.99,177.66) | -21.40 |
| Panama | 42.99 (32.97,52.94) | 54.07 (42.19,67.47) | 25.77 |
| Papua New Guinea | 66.73 (50.33,83.75) | 64.9 (49.03,82.08) | -2.74 |
| Paraguay | 28.9 (22.36,36.37) | 30.78 (24.06,38.97) | 6.51 |
| Peru | 35.15 (27.1,43.19) | 44.72 (34.66,56.12) | 27.23 |
| Philippines | 96.04 (73.16,119.13) | 110.34 (84.47,138.23) | 14.89 |
| Poland | 124.99 (96.24,154.32) | 162.27 (124.53,201.12) | 29.83 |
| Portugal | 90.86 (69.44,114.89) | 134.93 (103.44,174.59) | 48.50 |
| Puerto Rico | 56.52 (43.23,71.11) | 98.6 (74.53,126.3) | 74.45 |
| Qatar | 133.66 (99.48,174.48) | 109.37 (79.75,145) | -18.17 |
| Republic of Korea | 68.36 (51.69,85.67) | 134.59 (102.91,171.61) | 96.88 |
| Republic of Moldova | 105.09 (80.3,130.61) | 147.17 (113.26,184.73) | 40.04 |
| Romania | 117.99 (90.22,146.2) | 165.44 (127.71,209.84) | 40.22 |
| Russian Federation | 122.85 (93.77,152.56) | 144.51 (110.87,178.86) | 17.63 |
| Rwanda | 103.86 (80.94,126.77) | 106.95 (81.52,131.32) | 2.98 |
| Saint Kitts and Nevis | 54.05 (41.53,67.68) | 51.22 (38.75,65.27) | -5.24 |
| Saint Lucia | 45.26 (34.79,55.77) | 68.21 (51.93,86.92) | 50.71 |
| Saint Vincent and the Grenadines | 48.23 (36.99,59.61) | 65.1 (49.46,81.56) | 34.98 |
| Samoa | 58.37 (44.96,73.11) | 64.28 (49.48,80.63) | 10.13 |
| San Marino | 84.42 (64.57,107.4) | 120.47 (92.65,155.25) | 42.70 |
| Sao Tome and Principe | 120.84 (94.98,147.51) | 97.63 (74.52,121.2) | -19.21 |
| Saudi Arabia | 147.36 (113.48,185.41) | 119.94 (89.01,152.35) | -18.61 |
| Senegal | 113.74 (88.64,139.5) | 102.37 (78.75,125.15) | -10.00 |
| Serbia | 111.9 (85.09,140.38) | 150.83 (114.33,188.4) | 34.79 |
| Seychelles | 111 (86.23,137.88) | 121.76 (94.17,152.59) | 9.69 |
| Sierra Leone | 135.29 (105.96,164.85) | 105.03 (79.56,130.79) | -22.37 |
| Singapore | 73.71 (56.38,91.89) | 114.63 (88.5,144.02) | 55.51 |
| Slovakia | 110.68 (84.95,137.58) | 140.35 (107.94,177.47) | 26.81 |
| Slovenia | 115.88 (90.17,143.63) | 164.76 (125.43,210.25) | 42.18 |
| Solomon Islands | 63.89 (48.06,80.08) | 67.14 (50.63,83.54) | 5.09 |
| Somalia | 119.27 (93.49,147.91) | 113.89 (88.53,141.78) | -4.51 |
| South Africa | 70.02 (54.36,86.34) | 76.9 (59.25,95.52) | 9.83 |
| South Sudan | 118.68 (93.27,144.33) | 110.31 (85.99,136.15) | -7.05 |
| Spain | 92.33 (70.79,117.24) | 124.17 (94.77,159.38) | 34.48 |
| Sri Lanka | 106.34 (82.21,132.72) | 136.28 (104.63,169.28) | 28.15 |
| Sudan | 209.57 (164.9,260.81) | 143.26 (110.11,179.99) | -31.64 |
| Suriname | 45.79 (35.41,56.88) | 57.5 (44.28,71.93) | 25.57 |
| Sweden | 100.03 (76.77,126.91) | 104.45 (80.54,134.9) | 4.42 |
| Switzerland | 81 (62.31,103.15) | 100.69 (77.84,129.29) | 24.31 |
| Syrian Arab Republic | 170.18 (134.08,212.8) | 180.13 (139.25,226.12) | 5.85 |
| Taiwan (Province of China) | 44.09 (33.89,54.77) | 79.72 (61.49,101.82) | 80.81 |
| Tajikistan | 70.81 (54.88,87.5) | 70.93 (52.32,88.32) | 0.17 |
| Thailand | 108.19 (83.88,134.9) | 176.02 (136.14,220.99) | 62.70 |
| Timor-Leste | 97.8 (74.15,124.53) | 100.2 (76.96,125.59) | 2.45 |
| Togo | 99.6 (76.76,122.41) | 98.36 (75.2,122.63) | -1.24 |
| Tokelau | 93.63 (72.5,115.87) | 97.36 (75.48,120.47) | 3.98 |
| Tonga | 64.1 (49.37,80.26) | 72.02 (55.8,88.53) | 12.36 |
| Trinidad and Tobago | 43.05 (33.22,52.94) | 62.43 (47.87,79.39) | 45.02 |
| Tunisia | 196.27 (152.02,242.31) | 196.27 (151.32,240.95) | 0.00 |
| Turkey | 197.74 (153.83,245.58) | 192.06 (149.25,236.49) | -2.87 |
| Turkmenistan | 65.7 (50.19,81.38) | 78 (59.7,97.13) | 18.72 |
| Tuvalu | 79.79 (60.72,99.65) | 79.93 (60.84,100.29) | 0.18 |
| Uganda | 77.44 (60.33,94.62) | 63.51 (48.89,78.71) | -17.99 |
| Ukraine | 139.68 (107.28,175.06) | 166.83 (128.5,209.09) | 19.44 |
| United Arab Emirates | 126.33 (95.18,165.37) | 118.69 (84.64,156.61) | -6.05 |
| United Kingdom | 140.67 (108.93,176.92) | 146.47 (112.74,182.58) | 4.12 |
| United Republic of Tanzania | 83.53 (64.22,102.54) | 80.17 (62.36,98.88) | -4.02 |
| United States of America | 212.66 (166.9,264.18) | 250.29 (194.13,315.71) | 17.69 |
| United States Virgin Islands | 43.03 (33.11,53.8) | 89.66 (67.27,118.2) | 108.37 |
| Uruguay | 139.66 (109.23,173.92) | 161.57 (125.19,202.43) | 15.69 |
| Uzbekistan | 74.79 (57.88,91.9) | 78.98 (60.31,97.77) | 5.60 |
| Vanuatu | 60.49 (45.4,75.06) | 65.63 (49.6,82.51) | 8.50 |
| Venezuela (Bolivarian Republic of) | 37.65 (28.97,47.09) | 60.77 (46.83,75.34) | 61.41 |
| Viet Nam | 111.54 (86.69,139.28) | 125.84 (96.94,158.58) | 12.82 |
| Yemen | 192.88 (150.58,241.8) | 150.38 (116.57,189.74) | -22.03 |
| Zambia | 96.26 (74.46,116.72) | 84.38 (65.79,105.37) | -12.34 |
| Zimbabwe | 41.46 (32.06,51.2) | 39.04 (29.6,49.36) | -5.84 |
